# Cellular and Behavioral Functions of *fruitless* Isoforms in *Drosophila* Courtship

**DOI:** 10.1016/j.cub.2013.12.015

**Published:** 2014-02-03

**Authors:** Anne C. von Philipsborn, Sabrina Jörchel, Laszlo Tirian, Ebru Demir, Tomoko Morita, David L. Stern, Barry J. Dickson

**Affiliations:** 1 Research Institute of Molecular Pathology (IMP), Dr. Bohr-Gasse 7, 1030 Vienna, Austria; 2 Janelia Farm Research Campus, Howard Hughes Medical Institute, 19700 Helix Drive, Ashburn, VA 20147, USA

## Abstract

**Background:**

Male-specific products of the *fruitless* (*fru*) gene control the development and function of neuronal circuits that underlie male-specific behaviors in *Drosophila*, including courtship. Alternative splicing generates at least three distinct Fru isoforms, each containing a different zinc-finger domain. Here, we examine the expression and function of each of these isoforms.

**Results:**

We show that most *fru*^+^ cells express all three isoforms, yet each isoform has a distinct function in the elaboration of sexually dimorphic circuitry and behavior. The strongest impairment in courtship behavior is observed in *fru*^C^ mutants, which fail to copulate, lack sine song, and do not generate courtship song in the absence of visual stimuli. Cellular dimorphisms in the *fru* circuit are dependent on Fru^C^ rather than other single Fru isoforms. Removal of Fru^C^ from the neuronal classes vAB3 or aSP4 leads to cell-autonomous feminization of arborizations and loss of courtship in the dark.

**Conclusions:**

These data map specific aspects of courtship behavior to the level of single *fru* isoforms and *fru*^+^ cell types—an important step toward elucidating the chain of causality from gene to circuit to behavior.

## Highlights

• *fru* A, B, and C isoforms have largely overlapping expression in the male fly CNS
• All three *fru* isoforms contribute to male courtship, with *fru*^C^ being the most critical
• Fru^C^ specifies sexual dimorphisms in neuron number and arborizations
• Fru^C^ is required in defined neuronal classes for male-specific anatomy and behavior

## Teaser

*Drosophila* express three male-specific isoforms (A, B, and C) of the putative transcription factor *fruitless* (*fru*) in an overlapping pattern. von Philipsborn et al. show that isoform-mutant flies exhibit defects in male courtship, copulation success, and song production. Fru^C^ specifies sexual dimorphisms in defined neuronal classes potentially crucial for pheromone processing.

## Introduction

Males and females of sexually reproducing animal species typically display profound differences in their mating behaviors, reflecting the operation of sexually dimorphic neural circuits. Because most aspects of mating behaviors are innate, these sexual dimorphisms must be encoded in the genome and established during development. For several genetic model organisms, including flies and mice, the distinct behaviors of the two sexes and the initial molecular events that underlie sex determination are both well understood [1]. With the two endpoints thus well defined, the mating behaviors of these organisms provide an ideal opportunity to trace the long chain of causality from genes to behavior. This task involves defining the underlying neural circuitry at cellular resolution, relating specific cellular dimorphisms to specific behavioral dimorphisms, and understanding how these structural and functional dimorphisms are shaped by gene activity.

Progress toward this goal is currently most advanced for the male-specific courtship behavior of *Drosophila melanogaster* (reviewed in [2]). Sex in *Drosophila* is determined by the ratio of X chromosomes to autosomes. DNA-binding proteins have been identified that “count” chromosomes and trigger a cascade of gene regulatory events that results in female-specific expression of the *transformer* (*tra*) gene. *tra* determines almost all aspects of sexual differentiation, with the exception of the dosage compensation mechanisms that adjust expression levels of X-linked genes (reviewed in [3]). Thus, animals that are chromosomally female but lack *tra* function look and behave like males, whereas those that are chromosomally male but express *tra* look and behave like females [4]. The *tra* gene encodes a splicing factor with two known targets, *doublesex* (*dsx*) and *fruitless* (*fru*), both of which produce both male-specific (M) and female-specific (F) transcripts. *fru*^F^ transcripts appear to be nonfunctional, whereas *fru*^M^, *dsx*^M^, and *dsx*^F^ all encode predicted transcription factors essential for various aspects of sex-specific differentiation (reviewed in [5]).

The courtship behavior of *Drosophila* males consists of a series of discrete elements, including orientation toward the female, following the female, extending and vibrating one wing to produce a courtship song, licking the genitalia, and attempting copulation. Orientation, following, and singing are more common in the initial stages, whereas licking and attempted copulation are generally observed only during later stages of courtship with a sexually receptive female (reviewed in [6]). Multiple sensory inputs drive this behavior. Chemosensory and visual cues predominate, but neither is absolutely essential in single-pair assays performed under laboratory conditions. Chemosensory cues are thought to arouse the male and promote progression through courtship elements, whereas visual cues guide orientation and following [7].

Of the two distal genes in the sex determination pathway, *fru* plays the more prominent role in the establishment of sexually dimorphic neural circuitry and behavior. Males lacking *fru*^M^ appear to be externally normal males yet are profoundly defective in most aspects of courtship behavior [8–10]. Conversely, females engineered to express *fru*^M^ resemble normal females yet perform at least the initial stages of male courtship, albeit imperfectly [11, 12]. In contrast, the analogous mutations in *dsx* dramatically alter the animal’s appearance but have a comparatively milder impact on behavior, disrupting the song and reducing overall courtship levels [13, 14]. Tracing the causative links from genes to courtship behavior is thus most likely to be productive by following the *fru* branch of the sex determination pathway.

The sex-specific *fru* transcripts are expressed in ∼2,000 cells in the male nervous system [15], which have been subdivided on morphological and developmental criteria into ∼100 distinct classes and assembled into an anatomical atlas with cellular resolution [12, 16–18]. *dsx* is expressed in a subset of these cells [14, 19]. Neuronal silencing and activation experiments have demonstrated that the activity of the *fru*^+^ cells, collectively, is causally linked to the execution of courtship behavior [12, 16, 20]. Our working hypothesis is that many, perhaps even most, of these neurons contribute to some specific aspect of courtship behavior. For example, *fru*^+^ *Or67d*^+^ olfactory neurons detect the volatile inhibitory sex pheromone *cis*-vaccenyl acetate [21–23], *fru*^+^ *IR84a*^+^ olfactory neurons detect a plant volatile that stimulates courtship [24], and *fru*^+^ *ppk23*^+^ gustatory neurons detect the nonvolatile female aphrodisiac pheromone 7,11-heptacosadiene [25, 26]. Each of these chemosensory cues is likely to be further processed by *fru*^+^ neurons in the CNS [17, 27, 28]. Central *fru*^+^ neurons that contribute to various aspects of courtship song have also been identified [29–31], including the brain neurons P1/pMP4 and pIP10 and the thoracic neurons dPR1, vPR6, and vMS11. P1 is a critical node in the courtship circuitry, as it is stimulated by direct physical contact with a female [29] and its activity is both necessary and sufficient for song production [30].

If *fru*^M^ expression indeed defines most of the neurons likely to have sex-specific functions in male courtship behavior, then the task now is to identify sexual dimorphisms within this circuit, understand how they contribute to sexually dimorphic information processing and behavior, and determine whether and how they are specified by *fru* itself. Studies using light microscopy have identified at least 12 distinct classes of *fru*^+^ neurons that are sexually dimorphic [16–18, 27, 32], and higher-resolution anatomical and physiological studies are likely to reveal many more. These differences include a few neuronal classes that are present in males but lacking in females, such as P1, pIP10, and vPR6, and several others that differ in cell numbers, projections, or arborizations, such as mAL/aDT2, aSP1, and aSP2. The existence of P1 in males but not females is attributable to *dsx* [33] and might explain why only males sing. Dimorphisms in some of the other cell types have been attributed to *fru* [27, 32, 34], but for most cellular dimorphisms, it is still unknown whether they depend on *dsx* or *fru*. Moreover, the behavioral significance of these dimorphisms remains obscure. Thus, for the most part, both the causes and consequences of anatomical dimorphisms among the *fru*^+^ neurons are unknown.

Mapping cellular dimorphisms to the *fru* gene is further complicated by its complex molecular architecture. In addition to the sex-specific transcripts, a set of common transcripts (*fru*^COM^) is also produced from transcription that is initiated at alternative promoters, bypassing the target sequence for Tra and hence escaping sex-specific splicing [9, 10]. These *fru*^COM^ transcripts have essential functions during early embryonic development but are not expressed in adults and do not appear to contribute to sexual differentiation of the adult nervous system [35]. More importantly, the *fru* gene is additionally subject to alternative splicing at its 3′ end, resulting in at least four distinct variants (A–D) of both the *fru*^M^ and *fru*^COM^ transcripts. The Fru^A–D^ isoforms each contain a distinct zinc-finger domain and thus potentially have distinct DNA-binding properties [9, 10, 36]. It is thus important to know for each cellular dimorphism in the *fru* circuit not only whether it is dependent upon *fru* itself, but also upon which isoform. Similarly, it is imperative to know which aspects of courtship behavior are dependent upon each of the *fru* isoforms.

This effort has been initiated with an analysis of a mutation affecting the *fru*^C^ isoform, which disrupts courtship song and copulation, serotonergic innervations of the male reproductive system, and the central projections of foreleg gustatory neurons [34, 37]. Our primary objective in this work was to extend this study to a systematic investigation of all four major *fru* splice variants, mapping their expression to each of the distinct classes of *fru*^+^ neurons and assessing which cellular dimorphisms and which aspects of courtship behavior depend on each isoform. To the extent that currently available knowledge and genetic tools would allow, we also sought to correlate the cellular and behavioral functions of each *fru* isoform.

## Results

### Most *fru* Neurons Express Three Fru^M^ Isoforms

**Figure 1:**
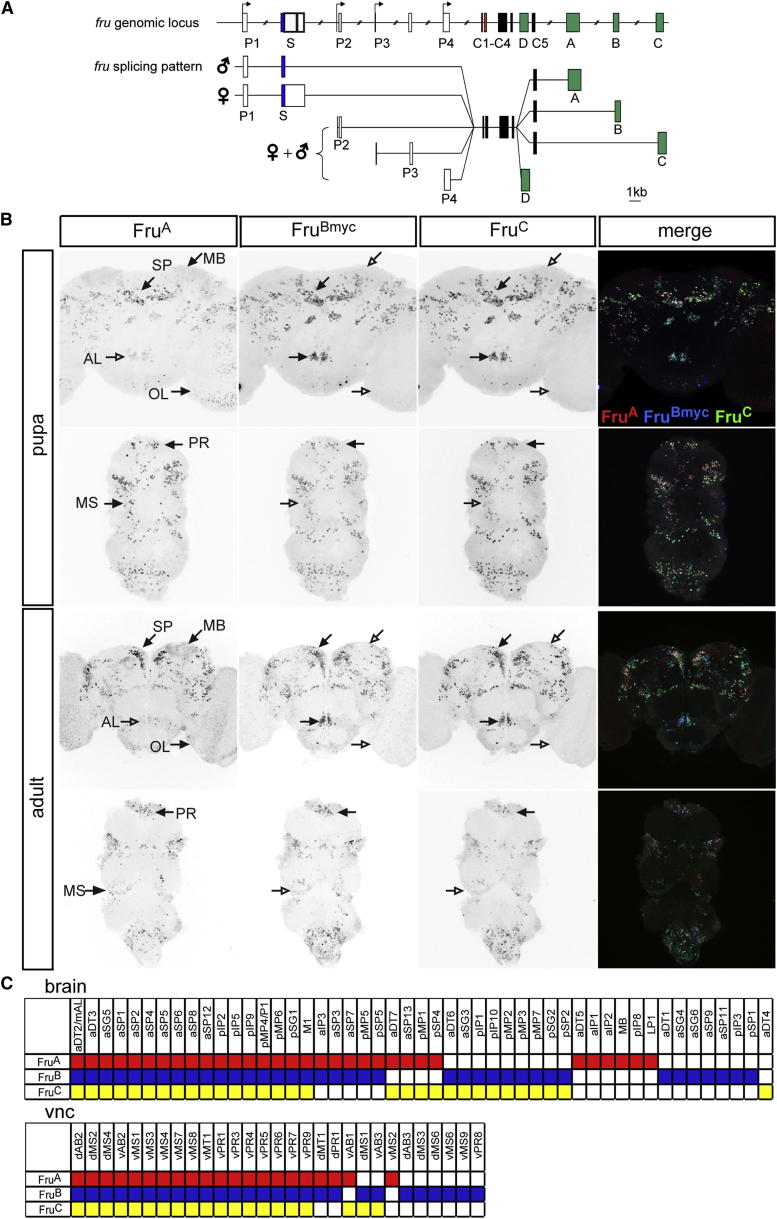
Overlapping Expression of Fru^M^ Isoforms in the Pupal and Adult CNS (A) Schematics of the *fru* genomic locus and splicing pattern. P1–P4, alternative promoters; S, sex-specifically spliced exon; C1–C5, common exons (encoding BTB domain); A–D, isoform-specific exons (encoding zinc-finger domains). *myc* tags were located at the 5′ end of isoform-specific exons A–D; exons A and C encode the respective antibody epitopes. (B) Brains and ventral nerve cords of male 48 hr pupa and 8 day adult flies triple labeled for Fru^A^, Fru^Bmyc^, and Fru^C^. Solid arrows indicate selected clusters showing expression of the indicated isoform; empty arrows the absence of staining. SP, medial superior protocerebrum; MB, mushroom body; AL, antennal lobes; OL, optic lobes; PR, ventral prothoracic ganglion; MS, mesothoracic ganglion. (C) Overlapping and distinct isoform expression of a set of 78 anatomically characterized adult neuronal classes. Expression of Fru^A^ is marked in red, Fru^B^ in blue, and Fru^C^ in yellow.

To determine the expression patterns of each of the four Fru^M^ isoforms, we used gene targeting by homologous recombination to independently attach c-*myc* epitope tags to the C termini of Fru^A^, Fru^B^, Fru^C^, and Fru^D^ (Figure 1A). The tagged Fru^A^, Fru^B^, and Fru^C^ proteins could be detected in the nuclei of most, but not all, *fru*^+^ neurons in the adult male brain and nerve cord (see Figure S1A available online; data not shown). Fru^D^ could not be detected in the adult male CNS (data not shown), consistent with the reported absence of *fru*^D^ mRNA in adult head tissue [9, 38]. As also expected, none of the isoforms could be detected in the adult female CNS (data not shown). In parallel, we generated antisera against protein domains encoded by the isoform-specific exons of Fru^A^ and Fru^C^ (Figure 1A) and confirmed their specificity by expressing *fru*^MA^, *fru*^MB^, and *fru*^MC^ transgenes in larval CCAP neurons (Figure S1B). Applied to brains of the corresponding c-*myc*-tagged *fru* allele, these antisera labeled the c-*myc*^+^ nuclei in males, with no staining detected in females (Figures S1C and S1D).

Using the specific antisera for Fru^A^ and Fru^C^ and the c-*myc*-tagged allele for *fru*^B^, we performed triple stainings to simultaneously visualize all three isoforms in the adult and pupal male CNS (Figure 1B). In broad agreement with previous reports [12, 16, 17], we counted 1,604 ± 123 cells expressing one or more Fru isoforms in the adult (n = 5) and 1,573 ± 89 cells in the pupa (n = 5). In both stages, we saw a high but not complete overlap in the expression of the three isoforms (Figure 1C; Table S1). We further mapped the expression of each of the three isoforms in 78 of the previously characterized [17, 30] *fru*^+^ neuronal classes (Figures 1C and S2). Most of these cells expressed all three Fru isoforms, but some expressed only one or two (Figure S2). Fru^A^ was detected alone in seven cell types, Fru^B^ in thirteen, and Fru^C^ in one (Figures 1B and S2).

### Isolation of Isoform-Specific *fru* Alleles

**Figure 2:**
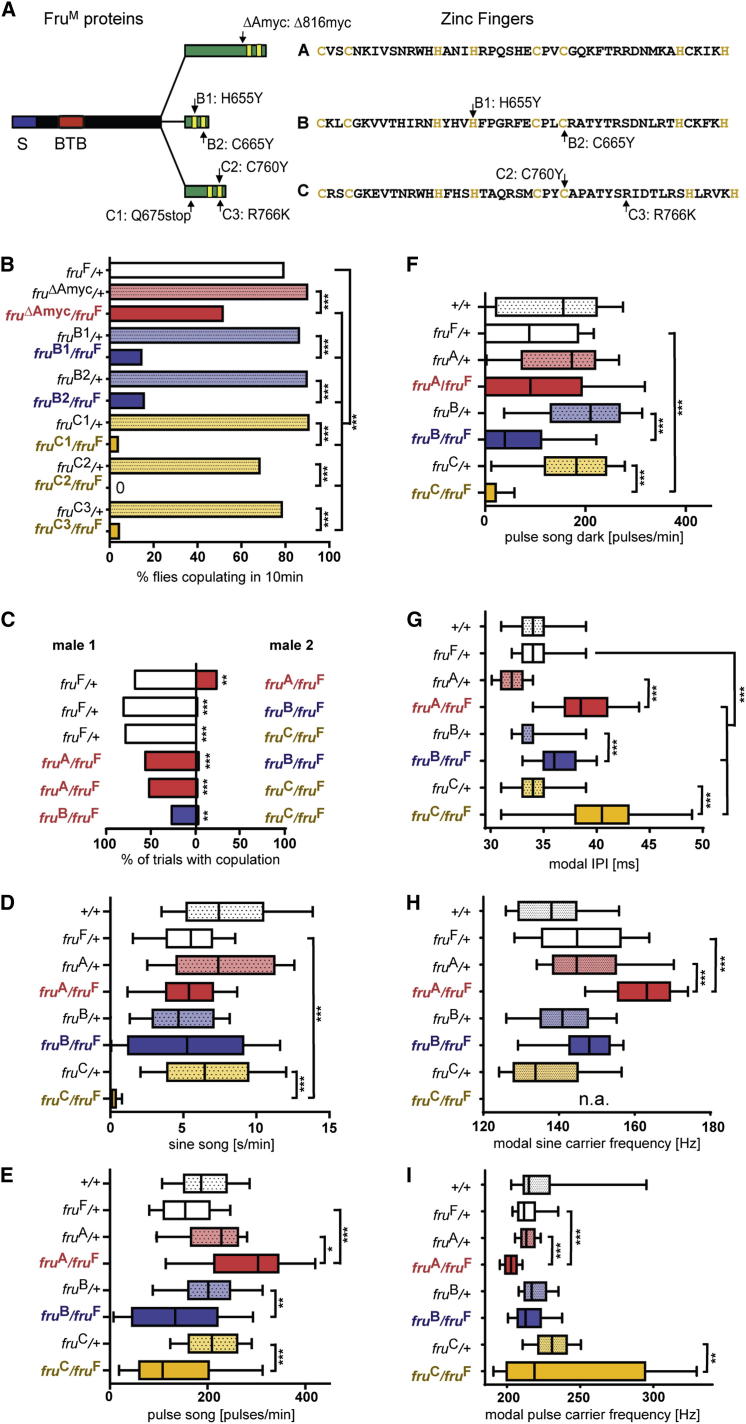
*fru* Isoform Mutants Display Distinct Impairments of Courtship Behavior (A) Fru^M^ proteins and mutations analyzed in this study. Sex-specifically spliced exon S is shown in blue, BTB domain in red, and isoform-specific domains with zinc fingers in green. Conserved cysteine and histidine residues in the zinc-finger sequences are shown in yellow. In the *fru*^ΔA^ allele, a myc sequence is placed after aa 816, replacing the C-terminal 139 residues. Nucleotide changes in the ethyl methanesulfonate-induced mutations are: *fru*^B1^, CAT→TAT at codon 655; *fru*^B2^, TGC→TAC at codon 665; *fru*^C1^, CAG→TAG at codon 675; *fru*^C2^, TGC→TAC at codon 760; and *fru*^C3^, AGG→AAG at codon 766. (B–I) Data for *fru*^A^ mutants are shown in red, for *fru*^B^ mutants in blue, and for *fru*^C^ mutants in yellow; the respective controls are shown in lighter color. Mutant genotypes (isoform-mutant alleles/*fru*^F^) are indicated in bold colored type; controls are indicated in regular black type. *fru*^A^ stands for *fru*^ΔAmyc^, *fru*^B^ for *fru*^B2^, and *fru*^C^ for the *fru*^C1^ isoform-mutant allele. (B) Copulation frequency of mutant males paired with wild-type virgin females in a 10 min courtship assay. n = 97–109 per genotype; ^∗∗∗^p < 0.0001 by Fisher’s exact test. (C) Competitive mating assay, with one male of each genotype competing for a single wild-type virgin female. Bars show the fraction of males copulating within 30 min. n = 26–40 pairs; ^∗∗∗^p < 0.0001, ^∗∗^p < 0.001 by Fisher’s exact test. (D–I) Box-and-whisker plots show 10^th^, 25^th^, 50^th^, 75^th^, and 90^th^ percentiles. ^∗∗∗^p < 0.0001, ^∗∗^p < 0.001, ^∗^p < 0.05 by Kruskal-Wallis nonparametric ANOVA followed by Dunn’s multiple comparisons test. (D) Amount of sine song. n = 50–75 flies per genotype. (E) Amount of pulse song. n = 50–75 flies per genotype. (F) Pulse song generation of mutant male flies in the dark. n = 72–75 flies per genotype. (G) Modal IPI in *fru* isoform mutants. n = 47–75 flies per genotype, 50–1,500 IPIs per fly. (H) Modal sine carrier frequencies. n = 40–64 flies per genotype. (I) Modal pulse carrier frequencies. n = 49–74 flies per genotype.

To assess the functions of each of the Fru isoforms, we next sought to generate a set of mutant alleles that selectively disrupt each of the Fru^A^, Fru^B^, or Fru^C^ isoforms. We first took advantage of the fact that *fru*^M^ and *fru*^Δtra^ are dominant sterile mutations in females [11]. We thus performed a chemical mutagenesis screen in the *fru*^Δtra^ background to isolate fertile intragenic revertants, a self-selecting phenotype. We recovered 16 revertant alleles in this manner, two of which were associated with mutations in the B exon (*fru*^B1^ and *fru*^B2^) and three in the C exon (*fru*^C1^, *fru*^C2^, and *fru*^C3^) (Figure 2A). The remaining alleles all carried mutations in the common exons. We did not recover any mutations in the A or D exons, presumably because these isoforms do not account for the sterility of *fru*^M^ or *fru*^Δtra^ females. We therefore targeted mutations to the A isoform directly, using homologous recombination to generate the *fru*^ΔA^ allele, in which the nucleotides encoding the predicted zinc-finger DNA-binding domain of the Fru^A^ specific exon are replaced by nucleotides encoding c-*myc* epitope tags (Figure 2A). In all of these alleles, the zinc-finger domain is predicted to be fully incapacitated or deleted (Figure 2A), and hence we attribute any phenotypic differences to different functions of each isoform rather than to different allele strengths. We also have not observed any dominant phenotypes associated with any of these alleles, further suggesting that the mutant proteins do not interfere with other molecular processes, for example by forming inactive complexes.

### Differential Contributions of Fru Isoforms to Mating Success and Courtship Song

The mutations in each of the isoform-specific *fru* alleles affect both the common and sex-specific transcripts. Indeed, like *fru* null mutants, both the *fru*^B^ and *fru*^C^ mutations are lethal in homozygotes (*fru*^ΔA^ homozygotes are viable without any obvious developmental abnormalities). To specifically assess the consequences of these mutations for the development and function of the courtship circuitry, we therefore examined males heterozygous for the *fru*^F^ allele and one of our isoform-specific alleles. In such males, the common transcripts (derived from the *fru*^F^ allele) retain the full isoform diversity, whereas the male-specific transcripts (derived from the isoform-specific allele) carry mutations in one of the zinc-finger domains. Hereafter, we refer to these *fru*^ΔA^/*fru*^F^, *fru*^B^/*fru*^F^, and *fru*^C^/*fru*^F^ males simply as *fru*^A^, *fru*^B^, and *fru*^C^ mutants. *fru*^A^, *fru*^B^, and *fru*^C^ mutants were fully viable and of normal size and did not show any obvious morphological abnormalities. *fru*^C^ mutants had reduced fertility, consistent with previous reports [37]. Fertility was also reduced in *fru*^B^ mutants, but not in *fru*^A^ mutants (data not shown).

In standard single-pair courtship assays, males mutant for any of the three isoforms still courted virgin females, but with reduced copulation success compared to wild-type control males (Figure 2B). *fru*^A^ mutants males were the least affected, and *fru*^C^ mutant males the most affected. There was no significant difference in performance between the two *fru*^B^ alleles *(fru*^B1^ and *fru*^B2^), nor between the two *fru*^C^ alleles (*fru*^C1^, *fru*^C2^, and *fru*^C3^). We therefore focused subsequent analyses on just one allele for each isoform, *fru*^B2^ and *fru*^C1^, respectively.

The relative mating deficits of each allele were confirmed in a series of competitive mating assays in which we pitted two males against each other in chambers with a single wild-type female. In these assays, *fru*^A^ males lost to *fru*^+^ (*fru*^F^*/+*) in about 75% of the cases but almost always outcompeted *fru*^B^ or *fru*^C^ males. *fru*^B^ males always lost against *fru*^+^ males but mostly outcompeted *fru*^C^ males. Finally, *fru*^C^ males always lost, regardless of opponent. Thus, the overall mating ability retained in each of these alleles can be ranked: *fru*^+^ > *fru*^A^ > *fru*^B^ > *fru*^C^ (Figure 2C).

One of the critical determinants of a male’s mating success is his courtship song, which consists of two distinct components, sine and pulse song. Sine song is a continuous 120–170 Hz vibration at low amplitude. Pulse song is a train of higher-amplitude 150–250 Hz pulses spaced at ∼35 ms intervals (the interpulse interval, or IPI). To assess courtship performance of isoform-mutant males in more detail, we quantified the amount and structure of the songs they produced. Sine song was normal for both *fru*^A^ and *fru*^B^ but almost completely absent in *fru*^C^ mutants (Figure 2D). None of the isoform mutants produced significantly less pulse song than control males heterozygote for *fru*^F^ (*fru*^F^*/+*) (Figure 2E). In contrast, pulse song of *fru*^C^ mutants, but not *fru*^A^ or *fru*^B^ mutants, was dramatically reduced when the flies were deprived of visual cues (Figure 2F). For all three alleles, pulse songs had longer and more varied IPIs than those of control males (Figure 2G). The carrier frequencies of sine and pulse song also varied in an allele-specific manner: *fru*^A^ mutants had higher-frequency sine song and lower-frequency pulse song, *fru*^B^ mutants sang with normal carrier frequencies, and for *fru*^C^ the carrier frequency of pulse song was highly variable but not significantly different than that of *fru*^F^*/+* control males (Figures 2H and 2I). Each mutant allele thus results in a distinct spectrum of song deficits.

### Differential Contributions of Fru Isoforms to Sexual Dimorphisms within the Courtship Circuit

**Figure 3:**
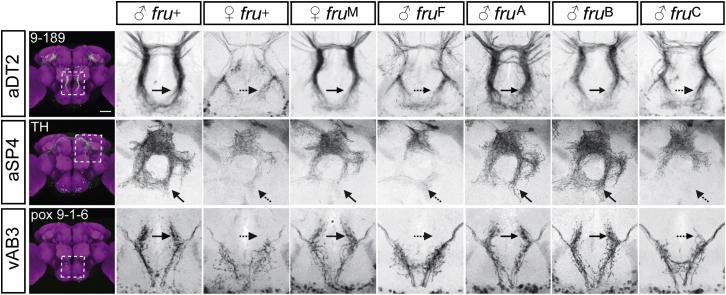
Fru^C^ Specifies Sexual Arborization Patterns Anatomical dimorphisms in the neuronal classes aDT2/mAL, aSP4, and vAB3. Each dimorphism was analyzed in the genetic backgrounds *fru*^+^, *fru*^M^, *fru*^F^, *fru*^ΔAmyc^, *fu*^B2^, and *fru*^C1^, in each case in combination with *fru*^FLP^, the GAL4 driver line indicated in the leftmost panel, and a *UAS>stop>mCD8-GFP* reporter to label the neurons of interest. Leftmost panels show registered confocal images of *fru*^+^ male brains stained with anti-GFP (green) and nc82 (magenta). Other panels are higher-magnification views of the approximate region indicated by the dashed box, showing averaged projections of 7–10 registered brains per genotype, stained with anti-GFP (black) and nc82 (for registration, not shown). Arrows indicate sex-specific arborizations (solid arrow, male type; dotted arrow, female type).

We next asked how each of the *fru*^A^, *fru*^B^, and *fru*^C^ mutations impairs the cellular substrate for courtship behavior, the *fru* circuit. We focused our attention on ten dimorphic *fru*^+^ cell types, several of which have been linked to song production. For seven of these cell types, cell number differs between the two sexes (Table 1); two are present in equal numbers in both males and females but have distinct arborization patterns (Figure 3), and one class differs in both cell number and arborizations (mAL/aDT2; Table 1 and Figure 3). For each of these cell types, we counted cells and compared their arborizations in wild-type males and females; *fru*^F^ males and *fru*^M^ females; and *fru*^A^, *fru*^B^, and *fru*^C^ mutant males.

As reported previously [33], the presence of P1/pMP4 neurons in males but not females is independent of *fru* (*fru*^F^ males have as many P1 cells as *fru*^+^ males; *fru*^+^ and *fru*^M^ females have none; Table 1). In contrast, for all other cell types that differ in number, this difference was either completely (pIP10, dPR1, vPR1, mAL/aDT2, aSP1, and aSP2) or partially (vPR6) dependent on *fru*. All of these cell types express multiple Fru isoforms, and for most of them we saw no differences in cell number in any of the *fru*^A^, *fru*^B^, and *fru*^C^ mutants. The two exceptions were vPR6, with fewer cells in *fru*^C^ mutants, and mAL/aDT2, for which slightly fewer cells were observed in both *fru*^B^ and *fru*^C^ mutants.

For each of the three cell types with dimorphic arborizations (mAL/aDT2, aSP4, and vAB3), we observed an apparently complete transformation to a male-like morphology in *fru*^M^ females and to a female-like morphology in *fru*^F^ males (Figure 3). These three cell types also each express multiple Fru isoforms, yet in each case, the male-specific arborization pattern was almost exclusively dependent upon *fru*^C^ function, with female-like arborizations observed in *fru*^C^ mutant males but relatively normal male-like arborizations in both *fru*^A^ and *fru*^B^ mutants (Figure 3).

### Cell-Specific Requirements for Fru^C^ for Male-Specific Anatomy and Behavior

**Figure 4:**
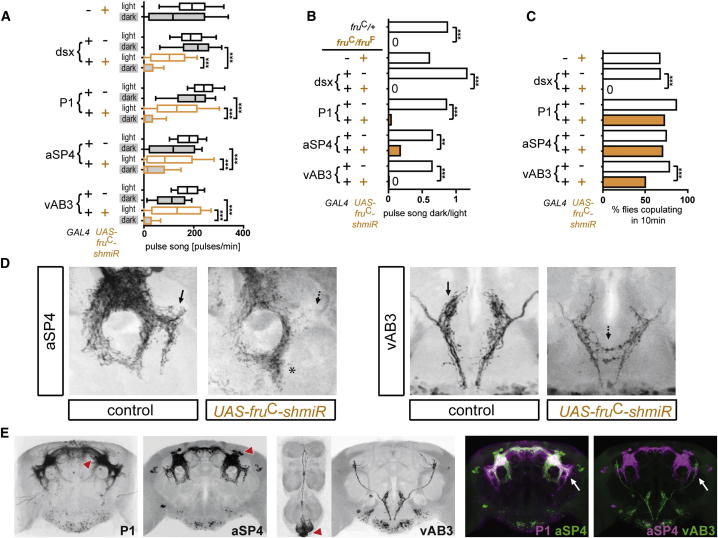
Cell-Specific Loss of Fru^C^ Impairs Courtship Behavior in the Absence of Visual Input (A) Pulse song production by males of the indicated genotypes, assayed in the light or dark in pairings with single wild-type virgin females. GAL4 lines used to target *fru*^C^ knockdown to the indicated cell types were *dsx-GAL4* (*dsx* neurons), *NP2631* (P1), *TH-GAL4* (aSP4), or *pox9-1-6-GAL4* (vAB3). n = 57–60 flies per genotype and condition. ^∗∗∗^p < 0.0001 by Kruskal-Wallis nonparametric ANOVA followed by Dunn’s multiple comparisons test. Box-and-whisker plots show 10^th^, 25^th^, 50^th^, 75^th^, and 90^th^ percentiles. In (A)–(C), data of flies with *UAS-fru*^C^*-shmiR*-mediated knockdown is plotted in orange, and control data are plotted in black and white; gray boxes in (A) indicate data recorded in the dark. (B) Ratio of median pulse song frequency in dark versus light conditions. ^∗∗∗^p < 0.0001, ^∗∗^p < 0.001 by permutation test (10,000 permutations). (C) Copulation frequency of males depleted of Fru^C^ in *dsx*, P1, aSP4, or vAB3 neurons, paired with wild-type virgin flies in a 10 min courtship assay. GAL4 lines used for knockdown were the same as in (A). n = 74–88 per genotype; ^∗∗∗^p < 0.0001 by Fisher’s exact test. (D) Registered projections of aSP4 and vAB3 in averaged images generated from 8–10 samples per genotype (*UAS-fru*^C^*-shmiR*, *TH-GAL4,UAS>stop>mCD8-GFP,fru*^FLP^ for aSP4 and *UAS-fru*^C^*-shmiR*, *pox1-9-6-GAL4,UAS>stop>mCD8-GFP,fru*^FLP^ for vAB3). Brain regions are shown as indicated in Figure 3, with solid and dotted arrows indicating male- and female-type arborizations, respectively. Asterisk indicates a GFP signal likely belonging to a neuronal type other than aSP4 (observed only after the prolonged aging required to detect the GFP signal in this genotype). (E) Arborization areas of P1, aSP4, and vAB3, depicted in average images of male brains and ventral nerve cords (n = 4–10 registered brains) from flies expressing GFP in *fru* neurons under the control of *NP2631*, *TH*, or *pox9-1-6*, respectively (*GAL4,UAS>stop>mCD8-GFP,fru*^FLP^*)*. Merged images show contacts of arborizations; arrows indicate the lateral extension of aSP4 lost upon knockdown of Fru^C^ as depicted in (D). Red arrowheads indicate the position of cell bodies for neuronal classes P1, aSP4, and vAB3.

How do anatomical dimorphisms at the cellular level relate to behavioral dimorphisms at the organismal level? To begin to address this question, we needed to assess the behavioral consequences of selectively disrupting the function of a single *fru* isoform in specific cell types, leaving the rest of the *fru* circuit unperturbed. As our anatomical studies had revealed single-isoform requirements only for *fru*^C^, which was also the allele with the most profound behavioral deficits, we generated a short micro-RNAi construct to specifically disrupt *fru*^C^ function in various cell types (*fru*^C^-*shmiR*). We confirmed that targeted expression of *fru*^*C*^-*shmiR* removed Fru^C^, but not Fru^A^ or Fru^B^, from specific *fru* neurons in the male brain (Figure S3A). Loss of the Fru^C^ isoform in *dsx* neurons recapitulated the loss of courtship in the dark (Figures 4A and 4B), as well as the defect in copulation seen in *fru*^C^ mutant males (Figure 4C). One of the most prominent brain neuronal classes expressing both *fru*^C^ and *dsx* is P1. We found that removing Fru^C^ in P1 by expressing *fru*^C^-*shmiR* under the *NP2631* driver was sufficient to inhibit courtship in the dark (Figures 4A, 4B, and S3B) but did not affect copulation success in the light (Figure 4C).

We focused next on the aSP4 and vAB3 cells, which do not express *dsx* (data not shown), but for which we had observed a unique requirement for Fru^C^ in shaping their arborization patterns. Removal of Fru^C^ in either aSP4 or vAB3 by driving expression of *fru*^C^-*shmiR* with the *TH* and the *pox9-1-6* driver, respectively (Figure S3B), also resulted in a specific and strong loss of courtship in the dark (Figures 4A and 4B). Copulation success in the light was not affected by knockdown of Fru^C^ in *TH*^+^ neurons but was moderately reduced upon knockdown of Fru^C^ in *pox9-1-6*^+^ neurons (Figure 4C). Loss of Fru^C^ in *NP2631*^+^, *TH*^+^, or *pox9-1-6*^+^ neurons did not affect sine song production in the light. Consistent with the moderate reduction of pulse song, loss of Fru^C^ in *dsx*^+^ neurons slightly reduced sine song but did not abolish it completely (Figure S3C). IPI distributions of pulse song were normal in each of these cell-specific Fru^C^ knockdowns (Figure S3D), implying that these behavioral deficits in *fru*^C^ mutants must map to other cells.

When we targeted *fru*^C^-*shmiR* to either aSP4 or vAB3, using the *TH-GAL4* and *pox9-1-6-GAL4* drivers respectively, we observed the same feminization of their morphology that we had seen in the wholly mutant *fru*^C^ males (Figure 4D). Accordingly, we conclude that *fru*^C^-dependent masculinization of both aSP4 and vAB3 arborizations might be essential for pulse song production in the absence of visual input. In light of the similar behavioral consequences of depleting Fru^C^ in P1, aSP4, and vAB3, it is interesting to note that the arbors of P1 and aSP4 overlap extensively in the dorsal protocerebrum, including in a lateral extension that is targeted by the dorsal arbor of vAB3 (Figure 4E). Notably, in the case of aSP4, this lateral extension is lost upon knockdown of Fru^C^, suggesting that any connection between vAB3 and aSP4 is likely to be *fru*^C^ dependent (Figure 4E).

## Discussion

The primary goal of this study was to determine the expression pattern of each of the Fru isoforms (A–D) in the developing and adult male CNS, at cellular resolution, and to assess the contribution that each makes to both anatomical and behavioral dimorphisms. Such information is essential to the ultimate goal of understanding how *fru* sculpts the sex-specific neural circuitry, and hence the neural computations, that generate male courtship behavior. We confirmed previous reports that the D isoform is not expressed in the developing or adult CNS and did not examine this isoform further. Each of the other three isoforms is expressed in the CNS and makes some contribution to male courtship behavior.

Fru^A^, Fru^B^, and Fru^C^ are coexpressed in many of the *fru*^+^ neurons, although several cell types express only one or two of these isoforms. A similar pattern of substantial but incomplete overlap of Fru isoforms has also been observed in the embryonic nervous system [35]. Splicing at the 3′ end of the *fru* transcripts thus appears to be regulated in a cell-specific manner, independent of the sex-specific splicing that some transcripts undergo at their 5′ end.

Functionally, we found specific deficits in courtship behavior in flies lacking any one of the three isoforms. Thus, each isoform has some nonredundant contribution to courtship behavior and, presumably, the construction or function of the underlying circuitry. In general, however, the deficits observed upon eliminating just one isoform were relatively mild compared to those observed upon complete loss of the male-specific *fru* transcripts. All three isoform mutants performed male-female courtship, but with significantly reduced success. *fru*^C^ mutants were the most affected, and *fru*^A^ mutants the least. *fru*^C^ mutants showed two striking defects in courtship behavior: the complete loss of sine song, and a dramatic reduction in pulse song in the absence of visual stimuli. The loss of sine song, which accounts for approximately half of the total song in control flies, is consistent with the significant reduction of wing extension observed previously in *fru*^C^ mutants [37]. Deprived of visual cues, a male presumably becomes more reliant on volatile or contact pheromones [7]. Consistent with this, males unable to detect the female stimulatory pheromone 7,11-heptacosadiene show loss of courtship song in the dark, but not in the light [26, 39]. The similar defects in *fru*^C^ mutants suggest that Fru^C^ might also be essential for detection or processing of this pheromone or other nonvisual stimuli from the female.

At the cellular level, we found that almost all sexual dimorphisms within the *fru* circuit depend upon the function of *fru* itself. It has been shown previously that the absence of P1 neurons in females is due to *dsx* and not *fru* [33]. On the other hand, dimorphisms in the number of mAL/aDT2 neurons, the size of the glomerular targets of *Or67d*^+^ OSNs, and the terminal arborizations of DA1 PNs had all been attributed to *fru* function [16, 27, 32]. Our analysis thus confirms and extends the general observation that most cellular dimorphisms among the *fru*^+^ neurons are indeed dependent upon *fru*. Moreover, we noticed an interesting pattern in the requirement for specific isoforms for each of these dimorphisms. For those cell types that are present in both sexes but differ in their arborization patterns, the Fru^C^ isoform was strictly required. In contrast, for those cell types that differ in number, no single isoform was essential. This might reflect redundancy among the distinct target genes of each isoform, or among their distinct binding sites at common target genes. Alternatively, Fru might regulate cell birth or survival by a mechanism that is independent of its zinc-finger domain.

Finally, in addition to determining how *fru* establishes all of these cellular dimorphisms, we also need to understand the impact that each has on neural processing and behavior. A useful strategy here might be to individually feminize each cell type and ask how this perturbs behavior and, as the tools become available, the underlying physiological processes. Here, we found that loss of Fru^C^ specifically in either aSP4 or vAB3 neurons feminizes their morphology and, we infer, changes their connectivity with pre- and postsynaptic partners. This might include connections between these two neurons, as aSP4 has Fru^C^-dependent lateral arborizations that overlap with the processes of vAB3. Depleting Fru^C^ from aSP4 or vAB3 recapitulates one aspect of the courtship defect observed in mutants that lack Fru^C^ entirely: the pronounced loss of pulse song in the dark. The appropriate morphology and connectivity of aSP4 and vAB3 might therefore be essential for male-specific processing of pheromone signals. From their anatomy, both aSP4 and vAB3 appear to be candidate input neurons for P1, which is activated by the female gustatory pheromone [29].

As further cellular dimorphisms within the *fru* circuit are revealed, the tools generated here can be used to assess the genetic determinants and behavioral consequences of these dimorphisms. These analyses will guide the formulation of hypotheses about the role of specific cellular dimorphisms in the information processing that occurs within these circuits—hypotheses that can be readily tested as the physiological methods for circuit analysis advance. It may then ultimately be possible to establish mechanistic links from a single gene to the complex behavior that it specifies, encompassing the expression of the specific Fru isoforms and the target genes they regulate, the cellular properties these genes influence, the circuit-level information processing these properties enable, and ultimately the probabilistic mapping of sensory input to motor output that characterizes courtship behavior in the fly.

## Experimental Procedures

### Fly Stocks

*UAS-lamin-GFP;fru*^GAL4^ were as described in [16]; *fru*^FLP^ and *UAS>stop>mCD8-GFP* were as described in [17]. *fru*^F^ and *fru*^M^ were as described in [11]. For validation of the isoform antibodies, *CCAP-GAL4* [40] was crossed to *UAS-Fru*^MA^, *UAS-Fru*^MB^, and *UAS-Fru*^MC^ [35]. Enhancer trap GAL4 lines (obtained from the Drosophila Genetic Resource Center, Kyoto Institute of Technology and the collection of U. Heberlein) were as described in [17]. The VT collection of molecularly defined enhancer GAL4 lines was generated using the strategy of [41] (C. Masser, S.S. Bidaye, A. Stark, and B.J.D., unpublished data). GAL4 lines driving expression in P1, aSP4, and vAB3 (*NP2631*, *TH-GAL4*, and *pox9-1-6*) were as described in [17]. These drivers are also expressed in one (*TH-GAL4* and *pox9-1-6*) or a few (*NP2631*) additional neuronal classes, none of which exhibited any morphological defects upon knockdown of Fru^C^. The strategy for targeting the *fru*^C^ transcripts was based on microRNA interference [42]. The targeting hairpin had the sequence ctagcagtCTGGCCATAAATCGCATCAGAtagttatattcaagcataTGTGATGCGAATTATGGCCAGgcg. For knockdown experiments, *w*^+^*;UAS-fru*^*C*^*-shmiR* virgins (CS background) were crossed to GAL4 lines with various genetic backgrounds.

### Gene Targeting

*fru*^Amyc^, *fru*^Bmyc^, *fru*^Cmyc^, and *fru*^Dmyc^ alleles were generated by ends-in homologous recombination [43], adding c-*myc* epitope tags to the carboxyl terminus of the Fru^A^, Fru^B^, Fru^C^, or Fru^D^ isoform. The donor construct contained a total of ∼7 kb of homology to the genomic sequence immediately upstream of the A, B, C, or D exon, with an I-SceI site approximately in the middle of the homology region. The homology region was followed by four in-frame c-*myc* epitope tags for *fru*^A^, *fru*^B^, and *fru*^C^ and two c-*myc* tags for *fru*^D^ (amino acid sequence EQKLISEEDLGS) without the deletion of endogenous sequences. A further ∼1.5 kb of homology following the endogenous stop codon, an I-CreI site, and the *white*^+^ marker were added. The duplication generated by ends-in targeting was removed by using *hsI-CreI* to introduce a double-stranded break at the I-CreI site and selecting progeny for the loss of the *white*^+^ marker. For generating the *fru*^ΔAmyc^ allele, the myc tag and stop codon were placed after codon G816 in the A exon (FBpp0083063; REFSEQ NP_732347), thereby deleting codons for the final 139 residues of Fru^A^. All targeted alleles were validated by sequencing genomic PCR products extending across the targeted region. The recombinant flies were backcrossed for five generations to a *w*^+^*;iso2;iso3* background prior to behavioral tests.

### *fru*^Δtra^ Reversion Screen

The *fru*^Δtra^ allele was marked by recombining it with a mini-white insertion on the third chromosome (*fru*^*Δ*tra^ *w*^*+*^). *fru*^Δtra^ *w*^*+*^/TM3 males were treated with ethyl methanesulfonate and crossed to *Ly,hs-hid*/TM3 virgins. The progeny were heat shocked during the late third instar. The eclosing males and females (∼85,000) were tested in small groups (ten females and males) for reversion of female fertility. Vials with more than ten pupae were kept, and the progeny were crossed inter se. Stocks were established from single males and tested for the presence of the *fru*^Δtra^ insertion, a *fru* allele by failure to complement the deficiency *Df(3R)Exel6179*, and the male-specific allele *fru*^*4-40*^. All protein-coding exons were sequenced from these revertants. The five alleles containing a mutation in either the B or C zinc-finger domain were backcrossed to an isogenic background (*w*^+^*;iso2;iso3*) for at least five generations prior to behavioral analysis.

### Antibody Generation

Fru^A^ and Fru^C^ antisera were obtained from rabbits and guinea pigs immunized with a GST fusion protein expressed from a cDNA containing the entire isoform-specific exon. The sera were purified against their antigen and dialyzed in PBS containing 50% glycerol. The reference sequences for the amino acids are FBpp0083063; REFSEQ NP_732347 for Fru^A^ and FBpp0083061; REFSEQ NP_732344 for Fru^C^.

### Behavioral Assays

Flies were raised on standard medium at 25°C in a 12:12 hr light: dark cycle and collected as virgins after eclosure. Females were kept in groups of up to 20, and males were housed individually. All behavioral experiments were conducted with 5- to 7-day-old males and 4- to 6-day-old females. For fertility tests, one male was kept with three females in a food vial that was checked after 4–5 days for progeny. Copulation frequency assays were performed in single-pair assays in chambers of 1 cm diameter for 10 min under constant light. Competitive courtship assays were carried out with an observation period of 30 min. The genotype of the competing males was distinguished by applying a terra cotta mark to the thorax at least 24 hr prior to testing. The mark itself did not affect courtship performance. Courtship song was recorded in beveled chambers of 11 mm diameter for 3.5 min or until copulation. The chambers were closed on one side with fine plastic mesh and placed on top of electret condenser microphones (CMP-5247TF-K, CUI Inc.). The signal was amplified with a custom-made circuit board and digitized with a multifunction data acquisition device (NI USB-6259 MASS Term, National Instruments) [44]. Tentative pulse and sine song were detected using a MATLAB script [44] and corrected manually in a custom-written user interface allowing fast annotation of sound oscillograms. For pulse/min counts, all pulses in trains of more than one pulse with IPIs of 15–150 ms were considered. For IPI analysis, all IPIs of 15–90 ms in trains of more than two pulses were considered. For IPI and carrier frequency analysis, only flies producing at least 50 of the respective events were considered. Mode values were determined by binning IPIs in 1 ms steps and carrier frequencies in 0.1 Hz steps. For comparing ratios of pulse song produced in the light versus in the dark, we employed a permutation test using a MATLAB script [45].

### Immunohistochemistry and Image Analysis

Fly dissection and staining were carried out as described previously [17]. Antibodies used were rabbit anti-GFP (1:6,000, Torrey Pines), chicken anti-GFP (1:3,000, Abcam), mouse mAb nc82 (1:20, Developmental Studies Hybridoma Bank), rabbit anti-Myc (1:12,000, Abcam), rat anti-Myc (1:8,000, Abcam), rabbit anti-Fru^A^ (1:4,000, see above), rabbit anti-Fru^C^ (1:4,000, see above), guinea pig anti-Fru^C^ (1:8,000, see above), rabbit anti-DsRed (to detect mCherry; 1:500 or 1:1,000, Clontech), and secondary Alexa 488, 568, and 647 antibodies (1:500 or 1:1,000, Invitrogen). Confocal stacks of stained brains and ventral nerve cords were taken on a Zeiss LSM 510 or a Zeiss LSM 700 with a Plan NeoFluar 25×/0.8 multi-immersion objective and analyzed with Amira software (Visage Imaging). Cell number analysis of Fru^M^ isoform expression in the CNS was carried out with Imaris software (Bitplane) using a cell diameter of 3.5 μm for spot detection and correcting manually for wrongly detected spots. Nonrigid image registration onto an nc82 standard template brain and image averaging were performed as described in [17].

## Figures and Tables

**Table 1:** Cell Number Dimorphisms

Neuronal Class	Cell Number Dependent on *fru*^M^?	Effect of Single Isoform?	Isoform Expression	♂ *fru*^+^	♀ *fru*^+^	♀ *fru*^M^	♂ *fru*^F^	♂ *fru*^ΔA^	♂ *fru*^B2^	♂ *fru*^C1^
pMP4/P1	no	no	A, B, C	17.3 ± 3.8 (n = 10)	0.0 ± 0.0 (n = 10)	0.0 ± 0.0 (n = 10)	17.3 ± 3.3 (n = 10)	17.7 ± 2.9 (n = 10)	17.9 ± 3.9 (n = 10)	17.1 ± 2.7 (n = 10)
pIP10	yes	no	B, C	0.7 ± 0.5 (n = 30)	0.0 ± 0.0 (n = 30)	1.0 ± 0.2 (n = 22)^∗∗∗^	0.0 ± 0.0 (n = 24)^∗∗∗^	0.8 ± 0.4 (n = 38)	1.0 ± 0.2 (n = 24)	0.9 ± 0.3 (n = 40)
dPR1	yes	no	A, B	0.9 ± 0.3 (n = 32)	0.0 ± 0.0 (n = 20)	0.8 ± 0.4 (n = 14)^∗∗∗^	0.0 ± 0.0 (n = 58)^∗∗∗^	0.9 ± 0.4 (n = 44)	0.8 ± 0.4 (n = 32)	0.9 ± 0.3 (n = 46)
vPR1	yes	no	A, B, C	3.1 ± 0.6 (n = 20)	0.2 ± 0.5 (n = 20)	2.8 ± 0.9 (n = 20)^∗∗∗^	0.6 ± 0.5 (n = 20)^∗∗∗^	3.2 ± 0.6 (n = 20)	3.1 ± 1.2 (n = 20)	2.8 ± 0.5 (n = 20)
vPR6	partially	yes, C	A, B, C	2.9 ± 0.7 (n = 18)	0.0 ± 0.0 (n = 20)	0.8 ± 0.8 (n = 16)^∗∗∗^	0.6 ± 0.9 (n = 20)^∗∗∗^	3.4 ± 0.6 (n = 20)	3.0 ± 0.8 (n = 18)	1.9 ± 1.0 (n = 20)^∗∗∗^
aDT2/mAL	yes	yes, B and C	A, B, C	11.1 ± 1.4 (n = 20)	5.1 ± 1.7 (n = 20)	12.3 ± 1.9 (n = 20)^∗∗∗^	5.1 ± 1.8 (n = 20)^∗∗∗^	10.2 ± 1.9 (n = 20)	8.1 ± 2.1 (n = 20)^∗∗∗^	8.2 ± 2.6 (n = 20)^∗∗∗^
aSP1	yes	no	A, B, C	13.3 ± 1.6 (n = 20)	2.2 ± 1.1 (n = 20)	14.5 ± 1.5 (n = 20)^∗∗∗^	5.0 ± 1.6 (n = 20)^∗∗∗^	15.0 ± 2.2 (n = 16)	14.9 ± 2.0 (n = 18)	15.2 ± 1.8 (n = 20)
aSP2	yes	no	A, B, C	62.1 ± 10.3 (n = 10)	32.9 ± 3.7 (n = 10)	63.0 ± 8.6 (n = 10)^∗∗∗^	34.3 ± 6.5 (n = 10)^∗∗∗^	67.0 ± 7.6 (n = 10)	67.6 ± 11.1 (n = 10)	62.9 ± 7.8 (n = 10)

Cell bodies per hemisphere ± SD. n, number of hemispheres evaluated. Asterisks indicate significant increase of cells in ♀ *fru*^M^ compared to ♀ *fru*^+^, significant decrease of cells in ♂ *fru*^F^ compared to ♂ *fru*^+^, and significant decrease of cells in ♂ *fru*^B2^ or ♂ *fru*^C1^ compared to ♂ *fru*^+^ (^∗∗∗^p < 0.0001, Mann-Whitney test).

## References

[1] Manoli D.S., Fan P., Fraser E.J., Shah N.M. (2013). Neural control of sexually dimorphic behaviors. Curr. Opin. Neurobiol..

[2] Yamamoto D., Koganezawa M. (2013). Genes and circuits of courtship behaviour in *Drosophila* males. Nat. Rev. Neurosci..

[3] Cline T.W., Meyer B.J. (1996). Vive la différence: males vs females in flies vs worms. Annu. Rev. Genet..

[4] Belote J.M., Baker B.S. (1987). Sexual behavior: its genetic control during development and adulthood in *Drosophila melanogaster*. Proc. Natl. Acad. Sci. USA.

[5] Billeter J.C., Rideout E.J., Dornan A.J., Goodwin S.F. (2006). Control of male sexual behavior in *Drosophila* by the sex determination pathway. Curr. Biol..

[6] Greenspan R.J., Ferveur J.F. (2000). Courtship in *Drosophila*. Annu. Rev. Genet..

[7] Krstic D., Boll W., Noll M. (2009). Sensory integration regulating male courtship behavior in *Drosophila*. PLoS ONE.

[8] Villella A., Gailey D.A., Berwald B., Ohshima S., Barnes P.T., Hall J.C. (1997). Extended reproductive roles of the *fruitless* gene in *Drosophila melanogaster* revealed by behavioral analysis of new *fru* mutants. Genetics.

[9] Ryner L.C., Goodwin S.F., Castrillon D.H., Anand A., Villella A., Baker B.S., Hall J.C., Taylor B.J., Wasserman S.A. (1996). Control of male sexual behavior and sexual orientation in *Drosophila* by the *fruitless* gene. Cell.

[10] Ito H., Fujitani K., Usui K., Shimizu-Nishikawa K., Tanaka S., Yamamoto D. (1996). Sexual orientation in *Drosophila* is altered by the *satori* mutation in the sex-determination gene *fruitless* that encodes a zinc finger protein with a BTB domain. Proc. Natl. Acad. Sci. USA.

[11] Demir E., Dickson B.J. (2005). *fruitless* splicing specifies male courtship behavior in *Drosophila*. Cell.

[12] Manoli D.S., Foss M., Villella A., Taylor B.J., Hall J.C., Baker B.S. (2005). Male-specific *fruitless* specifies the neural substrates of *Drosophila* courtship behaviour. Nature.

[13] Villella A., Hall J.C. (1996). Courtship anomalies caused by doublesex mutations in *Drosophila melanogaster*. Genetics.

[14] Rideout E.J., Dornan A.J., Neville M.C., Eadie S., Goodwin S.F. (2010). Control of sexual differentiation and behavior by the doublesex gene in *Drosophila melanogaster*. Nat. Neurosci..

[15] Lee G., Foss M., Goodwin S.F., Carlo T., Taylor B.J., Hall J.C. (2000). Spatial, temporal, and sexually dimorphic expression patterns of the *fruitless* gene in the *Drosophila* central nervous system. J. Neurobiol..

[16] Stockinger P., Kvitsiani D., Rotkopf S., Tirián L., Dickson B.J. (2005). Neural circuitry that governs *Drosophila* male courtship behavior. Cell.

[17] Yu J.Y., Kanai M.I., Demir E., Jefferis G.S.X.E., Dickson B.J. (2010). Cellular organization of the neural circuit that drives *Drosophila* courtship behavior. Curr. Biol..

[18] Cachero S., Ostrovsky A.D., Yu J.Y., Dickson B.J., Jefferis G.S.X.E. (2010). Sexual dimorphism in the fly brain. Curr. Biol..

[19] Robinett C.C., Vaughan A.G., Knapp J.M., Baker B.S. (2010). Sex and the single cell. II. There is a time and place for sex. PLoS Biol..

[20] Clyne J.D., Miesenböck G. (2008). Sex-specific control and tuning of the pattern generator for courtship song in *Drosophila*. Cell.

[21] Ha T.S., Smith D.P. (2006). A pheromone receptor mediates 11-cis-vaccenyl acetate-induced responses in *Drosophila*. J. Neurosci..

[22] Kurtovic A., Widmer A., Dickson B.J. (2007). A single class of olfactory neurons mediates behavioural responses to a *Drosophila* sex pheromone. Nature.

[23] van der Goes van Naters W., Carlson J.R. (2007). Receptors and neurons for fly odors in *Drosophila*. Curr. Biol..

[24] Grosjean Y., Rytz R., Farine J.-P., Abuin L., Cortot J., Jefferis G.S.X.E., Benton R. (2011). An olfactory receptor for food-derived odours promotes male courtship in *Drosophila*. Nature.

[25] Thistle R., Cameron P., Ghorayshi A., Dennison L., Scott K. (2012). Contact chemoreceptors mediate male-male repulsion and male-female attraction during *Drosophila* courtship. Cell.

[26] Toda H., Zhao X., Dickson B.J. (2012). The *Drosophila* female aphrodisiac pheromone activates *ppk23(*^+^) sensory neurons to elicit male courtship behavior. Cell Rep..

[27] Datta S.R., Vasconcelos M.L., Ruta V., Luo S., Wong A., Demir E., Flores J., Balonze K., Dickson B.J., Axel R. (2008). The *Drosophila* pheromone cVA activates a sexually dimorphic neural circuit. Nature.

[28] Ruta V., Datta S.R., Vasconcelos M.L., Freeland J., Looger L.L., Axel R. (2010). A dimorphic pheromone circuit in *Drosophila* from sensory input to descending output. Nature.

[29] Kohatsu S., Koganezawa M., Yamamoto D. (2011). Female contact activates male-specific interneurons that trigger stereotypic courtship behavior in *Drosophila*. Neuron.

[30] von Philipsborn A.C., Liu T., Yu J.Y., Masser C., Bidaye S.S., Dickson B.J. (2011). Neuronal control of *Drosophila* courtship song. Neuron.

[31] Pan Y., Robinett C.C., Baker B.S. (2011). Turning males on: activation of male courtship behavior in *Drosophila melanogaster*. PLoS ONE.

[32] Kimura K., Ote M., Tazawa T., Yamamoto D. (2005). Fruitless specifies sexually dimorphic neural circuitry in the *Drosophila* brain. Nature.

[33] Kimura K., Hachiya T., Koganezawa M., Tazawa T., Yamamoto D. (2008). Fruitless and doublesex coordinate to generate male-specific neurons that can initiate courtship. Neuron.

[34] Mellert D.J., Knapp J.-M., Manoli D.S., Meissner G.W., Baker B.S. (2010). Midline crossing by gustatory receptor neuron axons is regulated by fruitless, doublesex and the Roundabout receptors. Development.

[35] Song H.J., Billeter J.C., Reynaud E., Carlo T., Spana E.P., Perrimon N., Goodwin S.F., Baker B.S., Taylor B.J. (2002). The *fruitless* gene is required for the proper formation of axonal tracts in the embryonic central nervous system of *Drosophila*. Genetics.

[36] Dalton J.E., Fear J.M., Knott S., Baker B.S., McIntyre L.M., Arbeitman M.N. (2013). Male-specific Fruitless isoforms have different regulatory roles conferred by distinct zinc finger DNA binding domains. BMC Genomics.

[37] Billeter J.C., Villella A., Allendorfer J.B., Dornan A.J., Richardson M., Gailey D.A., Goodwin S.F. (2006). Isoform-specific control of male neuronal differentiation and behavior in *Drosophila* by the *fruitless* gene. Curr. Biol..

[38] Goodwin S.F., Taylor B.J., Villella A., Foss M., Ryner L.C., Baker B.S., Hall J.C. (2000). Aberrant splicing and altered spatial expression patterns in *fruitless* mutants of *Drosophila melanogaster*. Genetics.

[39] Lu B., LaMora A., Sun Y., Welsh M.J., Ben-Shahar Y. (2012). *ppk23*-Dependent chemosensory functions contribute to courtship behavior in *Drosophila melanogaster*. PLoS Genet..

[40] Kim Y.-J., Zitnan D., Galizia C.G., Cho K.H., Adams M.E. (2006). A command chemical triggers an innate behavior by sequential activation of multiple peptidergic ensembles. Curr. Biol..

[41] Pfeiffer B.D., Jenett A., Hammonds A.S., Ngo T.T., Misra S., Murphy C., Scully A., Carlson J.W., Wan K.H., Laverty T.R. (2008). Tools for neuroanatomy and neurogenetics in *Drosophila*. Proc. Natl. Acad. Sci. USA.

[42] Haley B., Hendrix D., Trang V., Levine M. (2008). A simplified miRNA-based gene silencing method for *Drosophila melanogaster*. Dev. Biol..

[43] Gong W.J., Golic K.G. (2003). Ends-out, or replacement, gene targeting in *Drosophila*. Proc. Natl. Acad. Sci. USA.

[44] Arthur B.J., Sunayama-Morita T., Coen P., Murthy M., Stern D.L. (2013). Multi-channel acoustic recording and automated analysis of *Drosophila* courtship songs. BMC Biol..

[45] Kamyshev N.G., Iliadi K.G., Bragina J.V. (1999). *Drosophila* conditioned courtship: two ways of testing memory. Learn. Mem..

