# Supplementary material for: Cellular and Behavioral Functions of fruitless Isoforms in Drosophila Courtship
Source: Curr Biol. 2014 Feb 3;24(3):242–51. doi: 10.1016/j.cub.2013.12.015 (PMC3969150; doi:10.1016/j.cub.2013.12.015)
Supplement: Document S1. Figures S1–S3 and Table S1 [file mmc1.pdf]

Current Biology, Volume *24*

Supplemental Information

Cellular and Behavioral Functions of *fruitless* Isoforms in *Drosophila* Courtship

Anne C. von Philipsborn, Sabrina Jörchel, Laszlo Tirian, Ebru Demir, Tomoko Morita, David L. Stern, and Barry J. Dickson

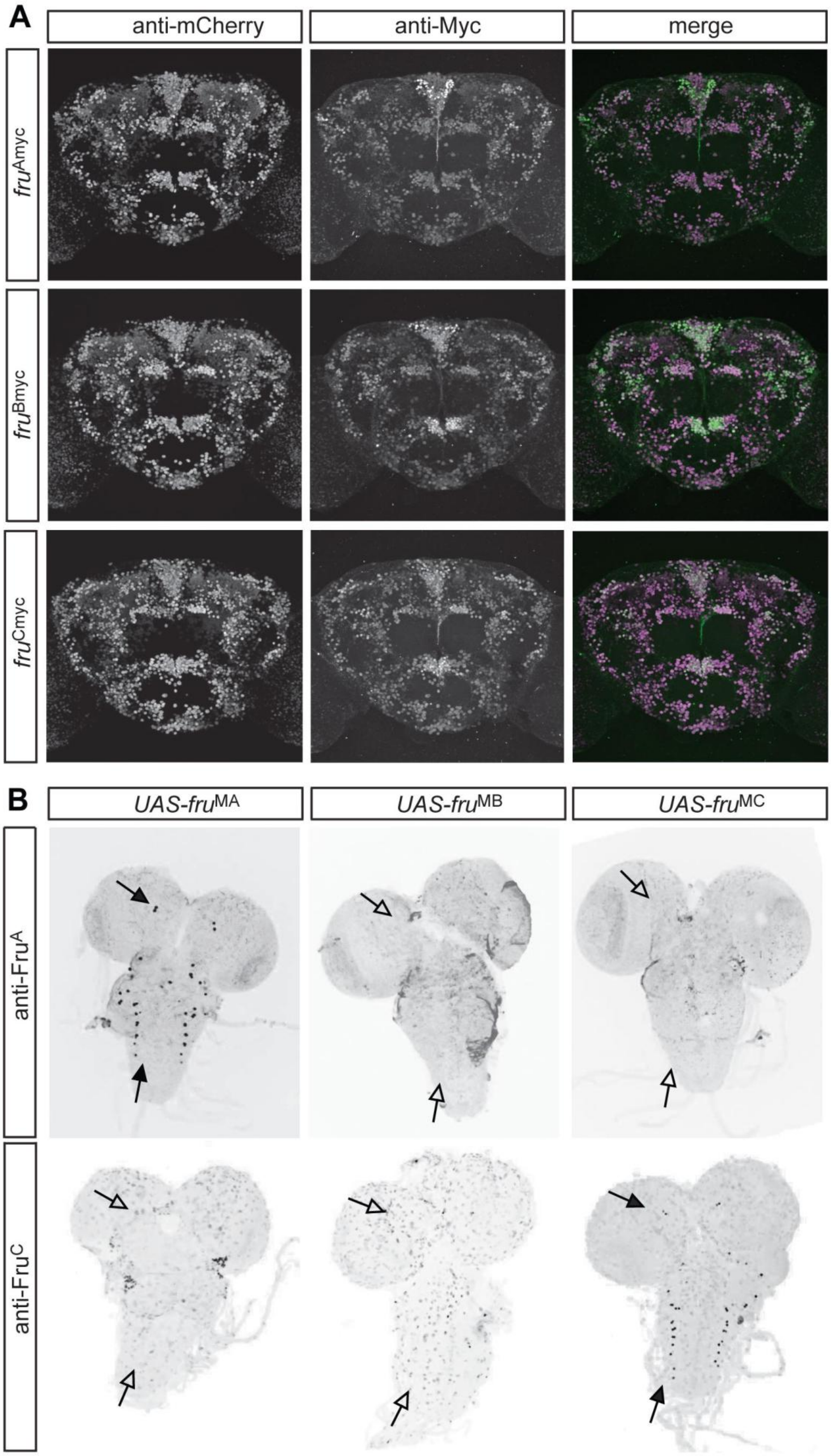
A
anti-mCherry
anti-Myc
merge
fru^{Amyc}
fru^{Bmyc}
fru^{Cmyc}
B
$UAS\text{-}fru^{MA}$
$UAS\text{-}fru^{MB}$
$UAS\text{-}fru^{MC}$
anti-Fru^{A}
anti-Fru^{C}

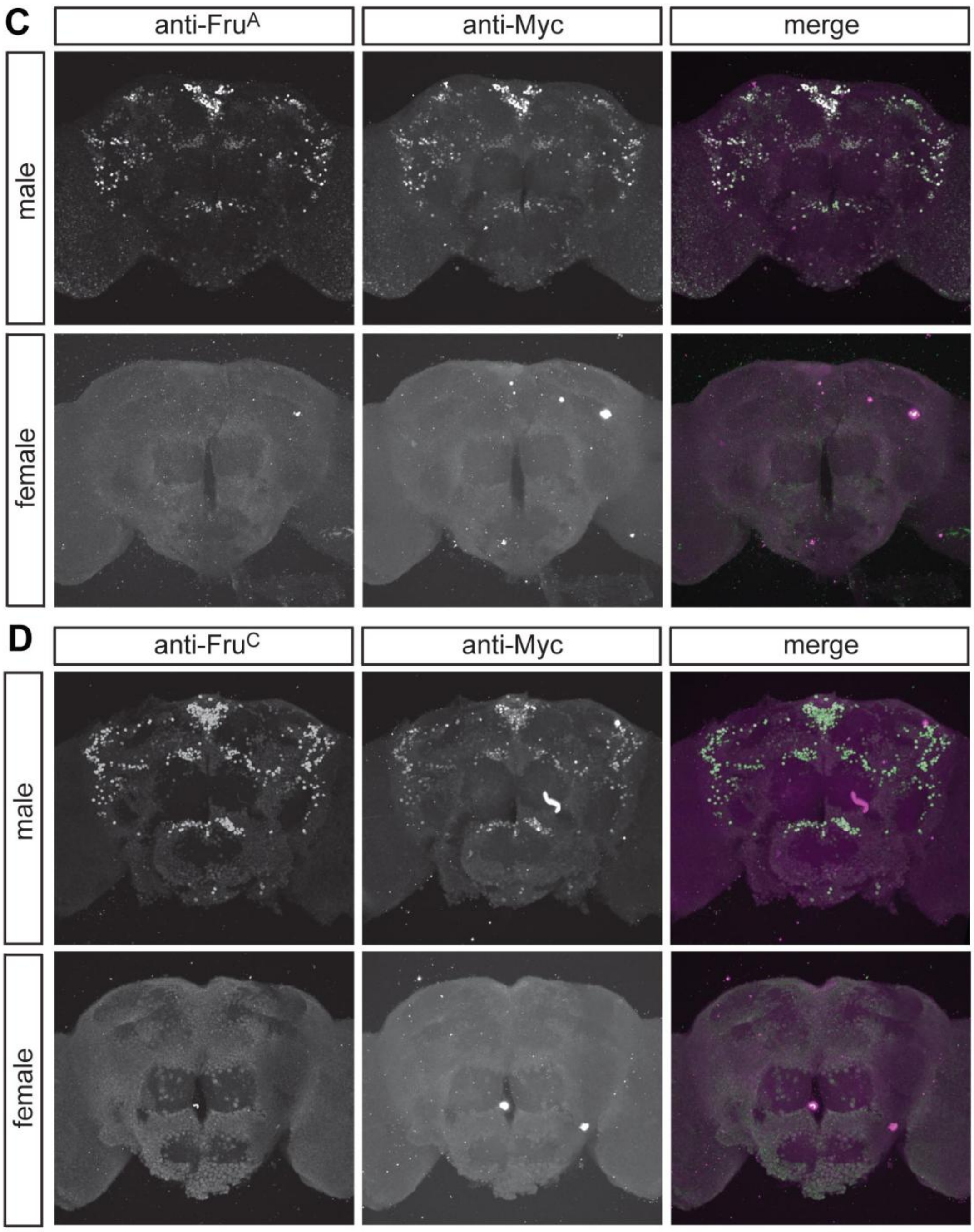

Figure S1, related to Figure 1

Validation of myc-tagged *fru* alleles and Fru^A and Fru^C antisera

A Brains of 7d-old males carrying *fru-GAL4, UAS-His2AmCherry* and the indicated fru^{myc} allele, stained with anti-mCherry (magenta in merge) and anti-c-Myc (green). **B** Brains and VNCs of 3rd instar larvae carrying *CCAP-GAL4* and the indicated UAS transgene, stained with anti-Fru^A or anti-Fru^C. Solid arrows indicate specific labeling in CCAP neurons; empty arrows the absence of labeling. Weak staining in CCAP-negative cells is likely due to expression of Fru^{COM} isoforms. **C, D** Brains of 7d-old adult males and females heterozygous for the fru^{Amyc} (C) or the fru^{Cmyc} (D) allele, stained with anti-Fru^A (C) or anti-Fru^C (D) (magenta) and anti-c-Myc (green).

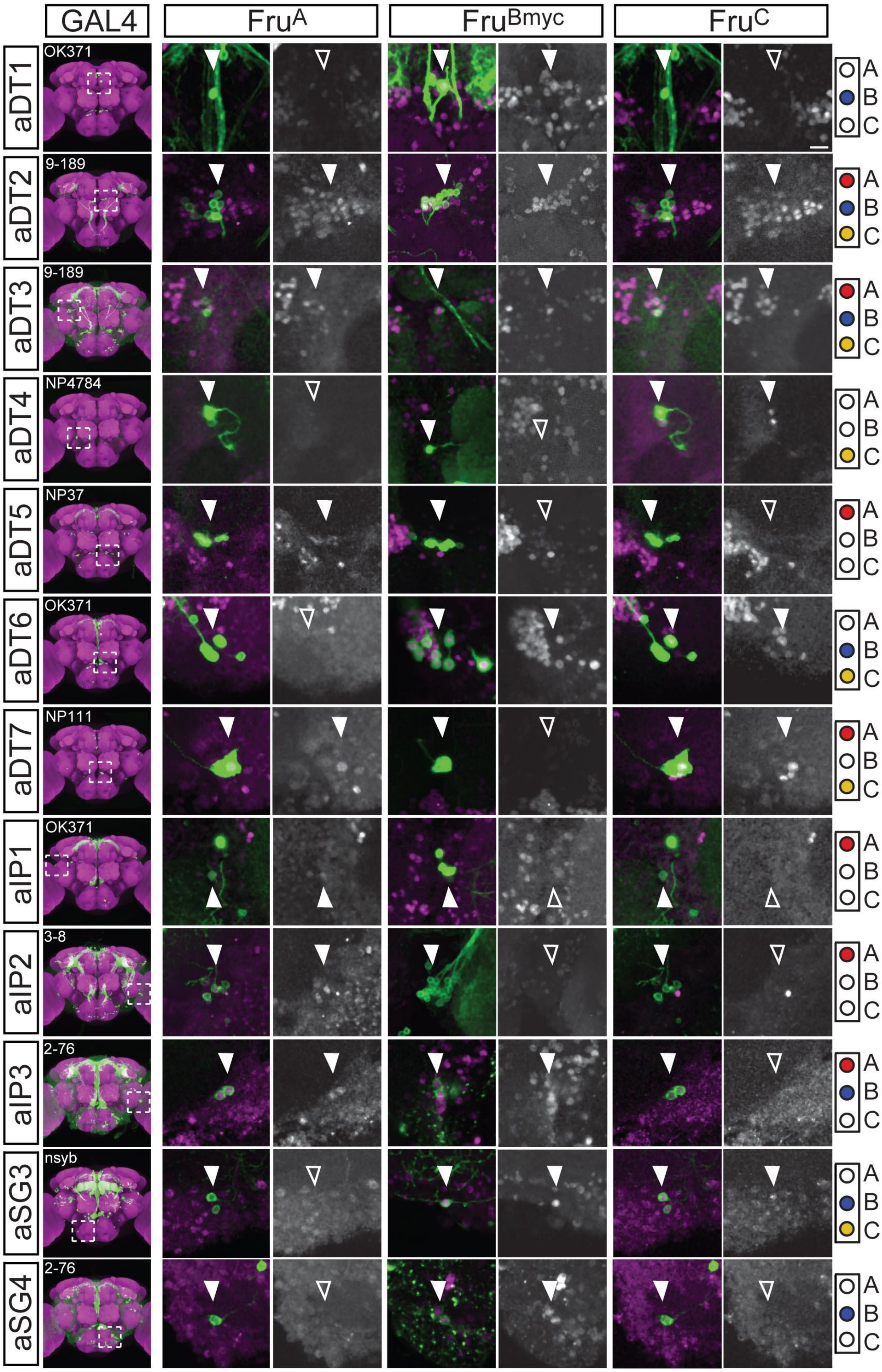
GAL4
Fru^A
Fru^Bmyc
Fru^C
aDT1
aDT2
aDT3
aDT4
aDT5
aDT6
aDT7
aIP1
aIP2
aIP3
aSG3
aSG4
OK371
9-189
9-189
NP4784
NP37
OK371
NP111
OK371
3-8
2-76
nsyb
2-76
A
B
C

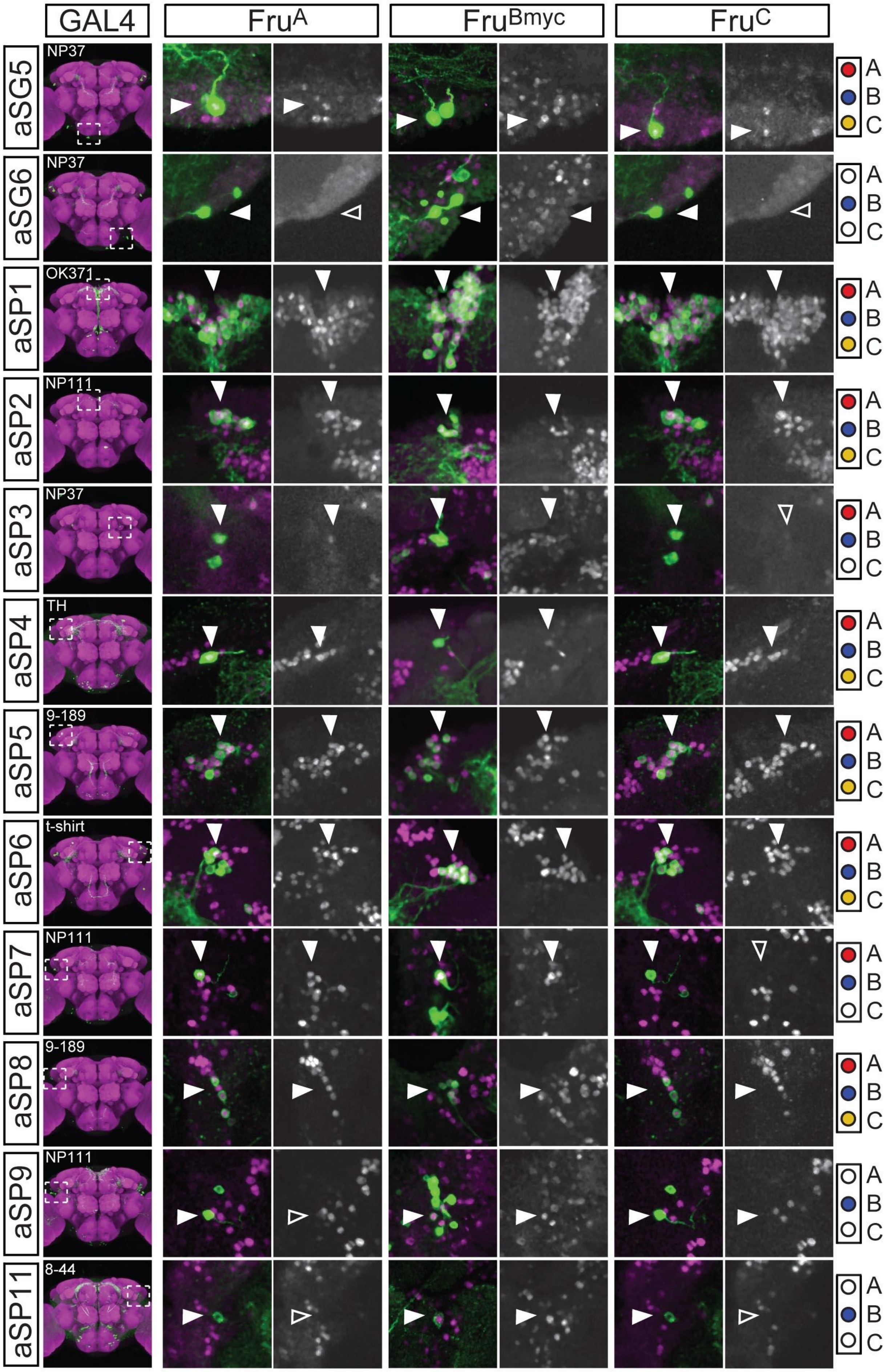

GAL4
Fru^A
Fru^Bmyc
Fru^C
aSG5
aSG6
aSP1
aSP2
aSP3
aSP4
aSP5
aSP6
aSP7
aSP8
aSP9
aSP11
NP37
OK371
NP111
TH
9-189
t-shirt
8-44
A
B
C

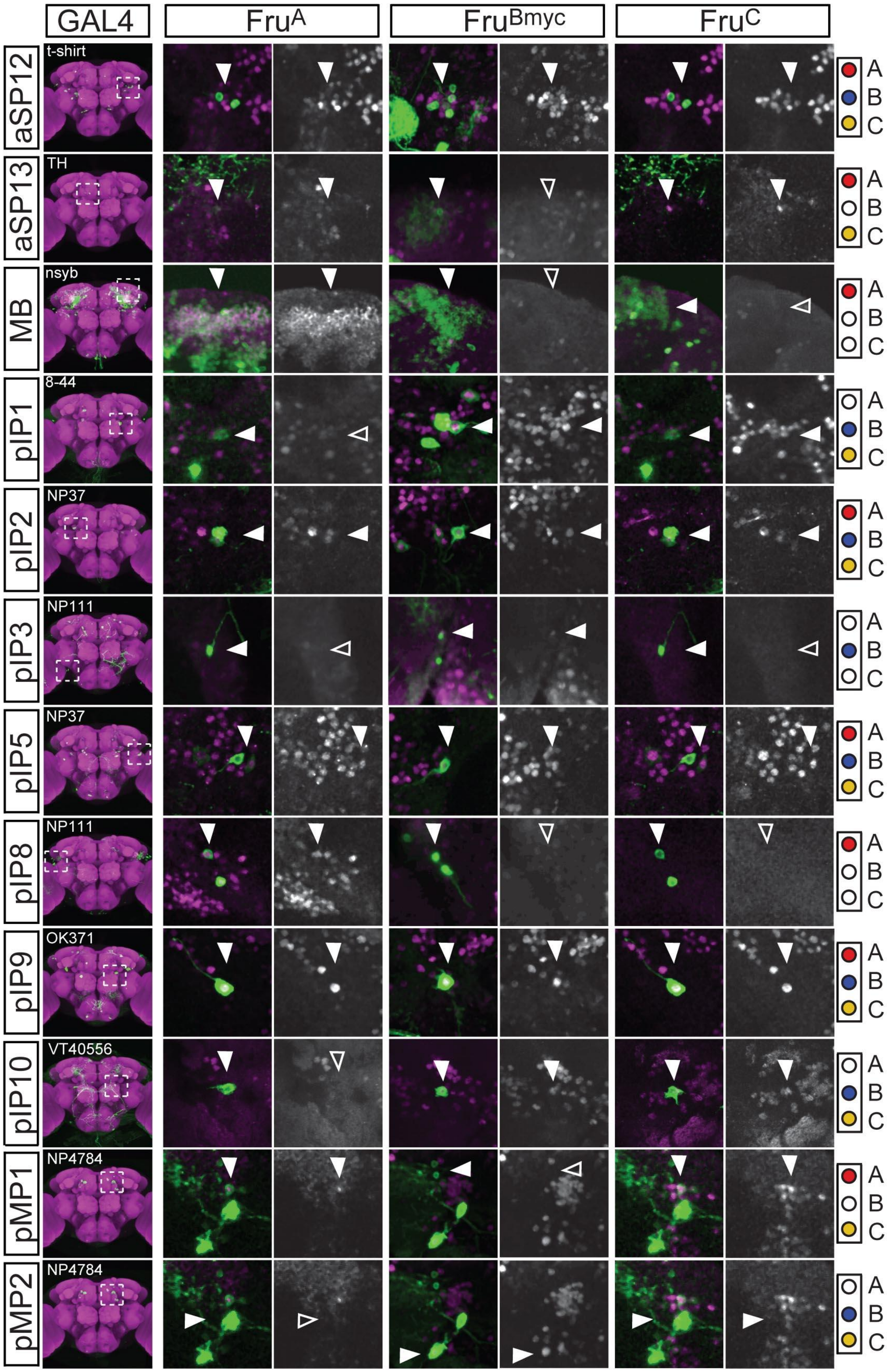

GAL4
Fru^A
Fru^Bmyc
Fru^C
aSP12
aSP13
MB
pIP1
pIP2
pIP3
pIP5
pIP8
pIP9
pIP10
pMP1
pMP2
t-shirt
TH
nsyb
8-44
NP37
NP111
NP37
NP111
OK371
VT40556
NP4784
NP4784
A
B
C

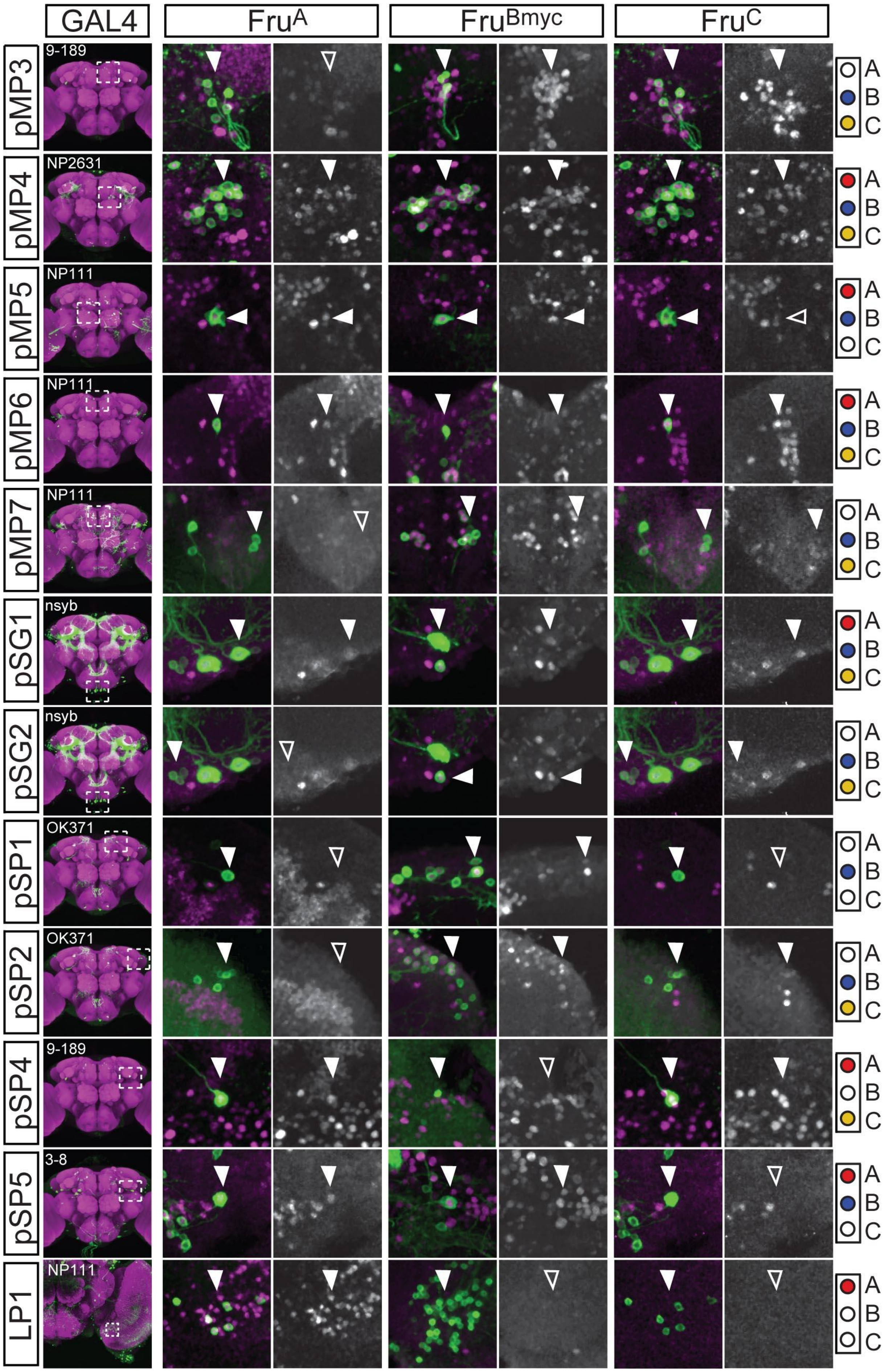

GAL4
FruA
FruBmyc
FruC
pMP3
9-189
pMP4
NP2631
pMP5
NP111
pMP6
NP111
pMP7
NP111
pSG1
nsyb
pSG2
nsyb
pSP1
OK371
pSP2
OK371
pSP4
9-189
pSP5
3-8
LP1
NP111
A
B
C

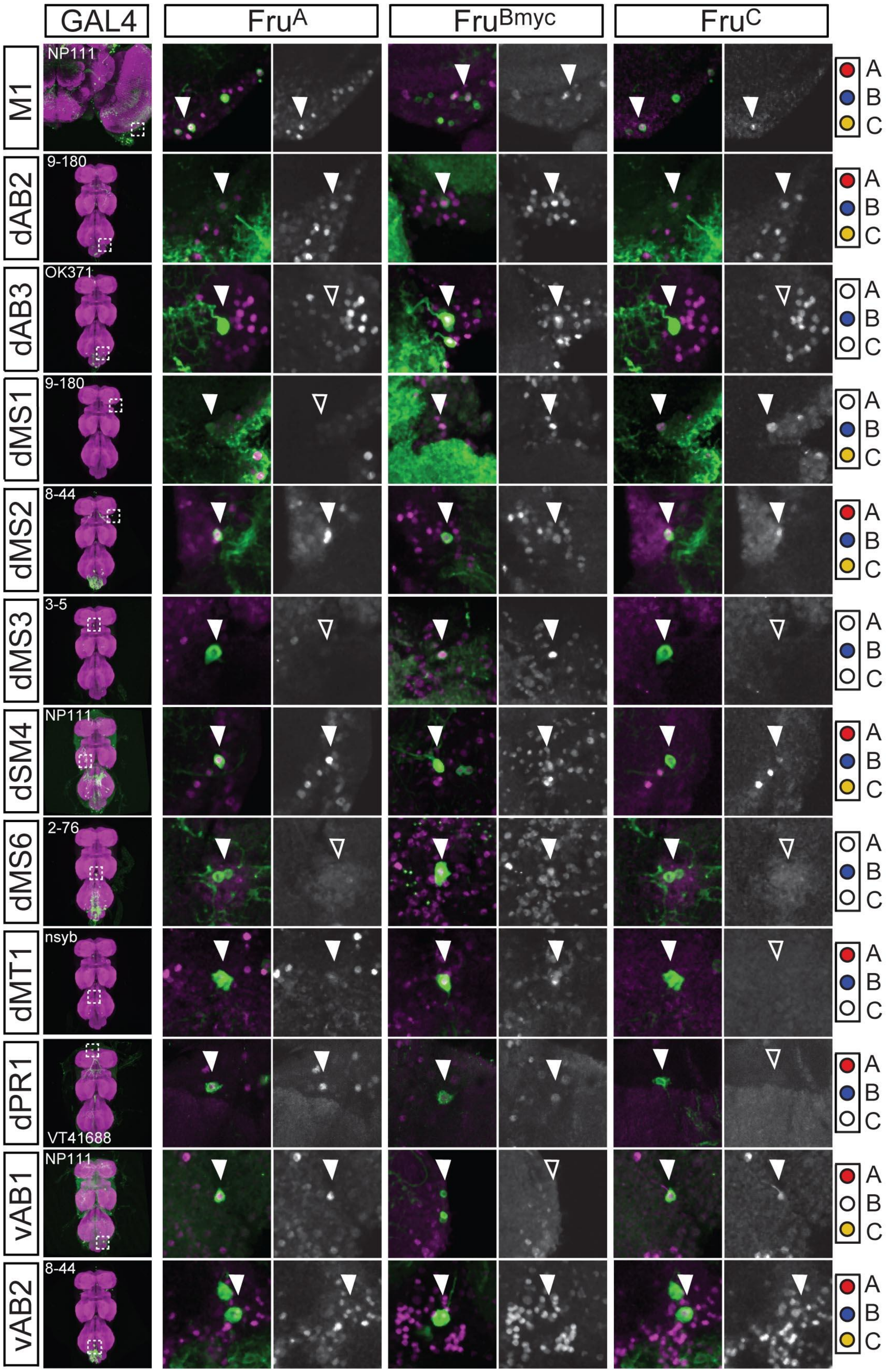

GAL4
FruA
FruBmyc
FruC
M1
NP111
dAB2
9-180
dAB3
OK371
dMS1
9-180
dMS2
8-44
dMS3
3-5
dSM4
NP111
dMS6
2-76
dMT1
nsyb
dPR1
VT41688
vAB1
NP111
vAB2
8-44
A
B
C

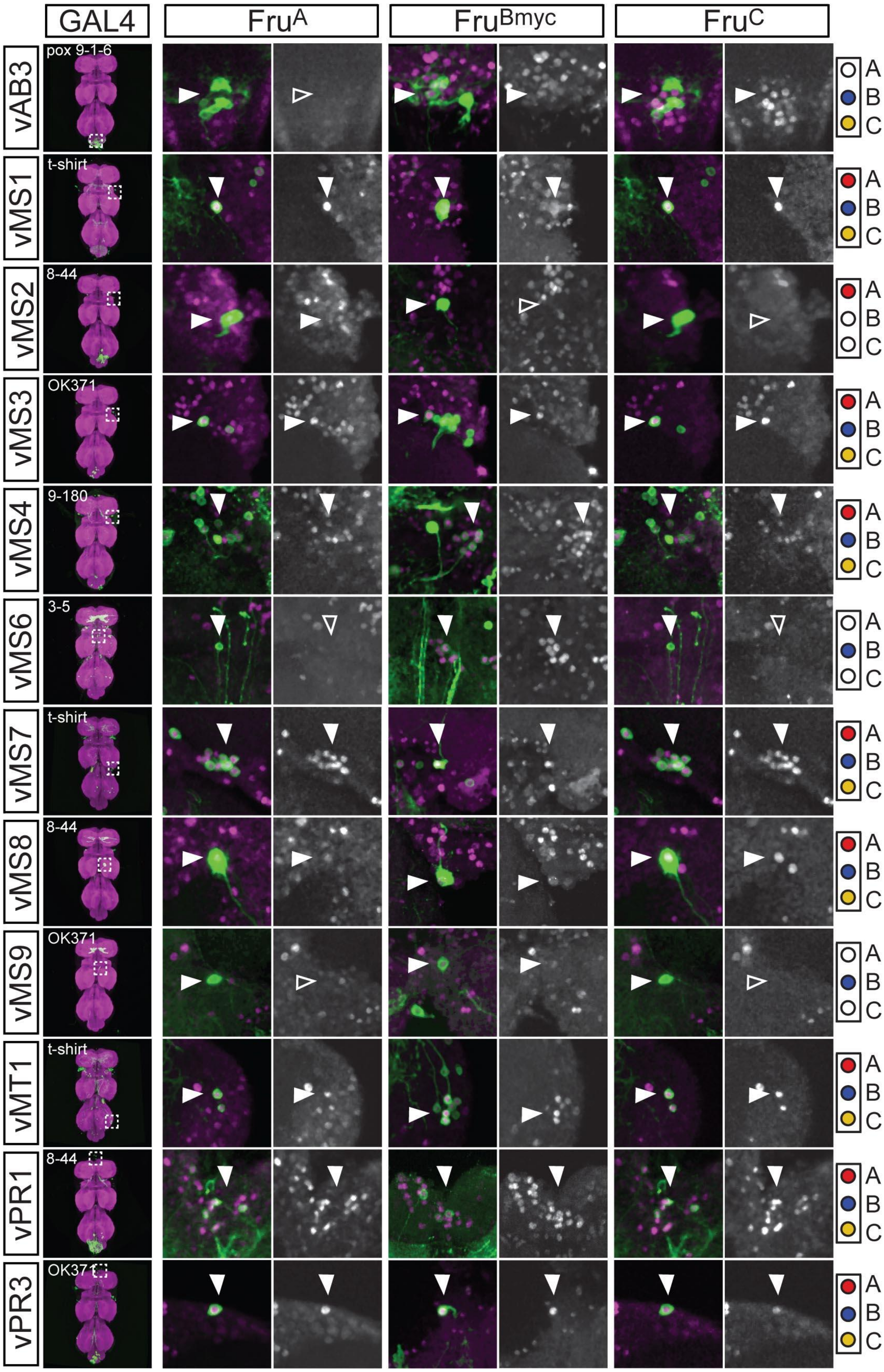

GAL4
Fru^A
Fru^Bmyc
Fru^C
vAB3
vMS1
vMS2
vMS3
vMS4
vMS6
vMS7
vMS8
vMS9
vMT1
vPR1
vPR3
pox 9-1-6
t-shirt
8-44
OK371
9-180
3-5
A
B
C

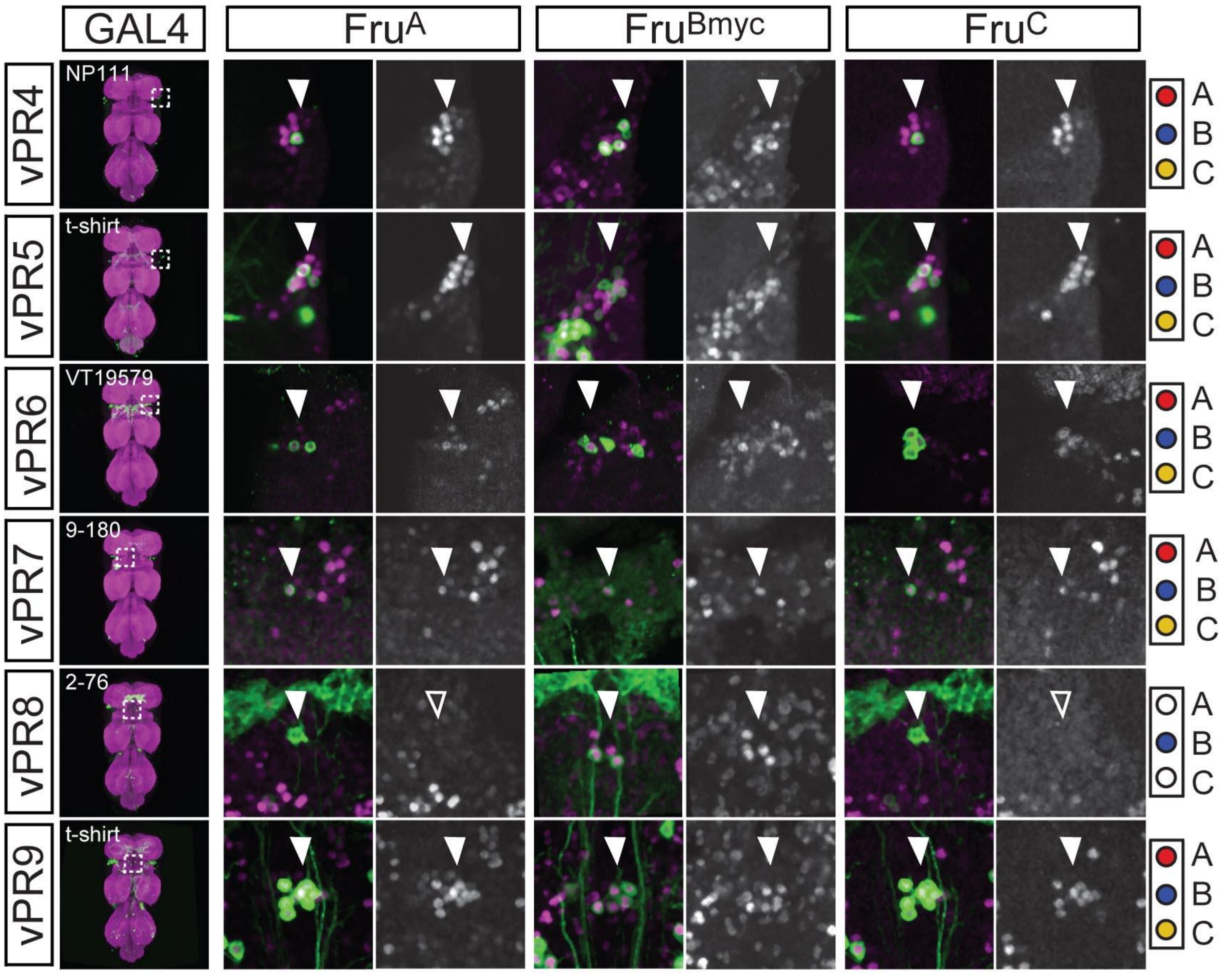

Figure S2, related to Figure 1

Expression of Fru isoforms in specific *fru*$^{+}$ cell types

For each cell type, the first column shows the location of the cell bodies (green) and indicates the *Gal4* line used to drive *UAS-mCD8-GFP* for identification. Selected confocal sections are shown registered onto a reference template (magenta). Fru^{A} and Fru^{C} were detected by antibody staining, Fru^{B} by use of the myc tagged allele and subsequent anti-Myc staining (shown in magenta). Solid and empty arrowheads mark the presence and absence of an isoform, respectively. The expression profile for each neuronal class is shown schematically in the rightmost panel. For detailed anatomical description and segmented projection and arborization patterns of the shown neuronal classes, see [17] and, for pIP10, dPR1 and vPR6, [30].

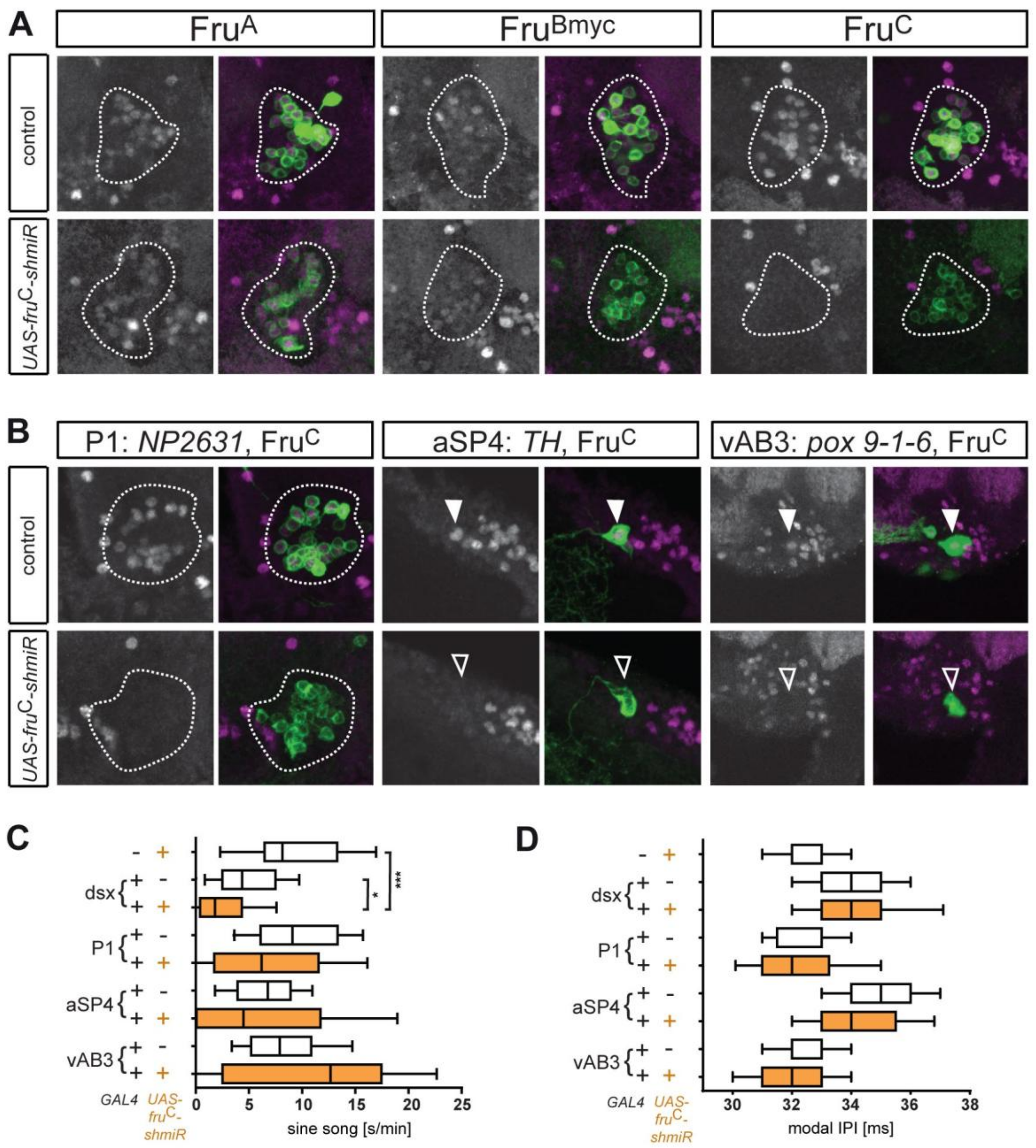

Figure S3, related to Figure 4

Knockdown of Fru^{C} in specific neuronal classes and its effect on courtship song

A *fru^{C} -shmiR* mediated knockdown leads to the depletion of Fru^{C}, but not Fru^{B} and Fru^{A}, as shown for the P1 neuronal cluster labeled by GFP expression driven by *dsx-GAL4* (green). Fru^{A} and Fru^{C} are labeled by isoform specific antisera, Fru^{B} by myc tagging and Myc antiserum (magenta). **B** Loss of Fru^{C} (detected by isoform specific antiserum, magenta) in the neuronal classes P1, aSP4 and vAB3 (labeled by GFP in green) after *fru^{C}-shmiR* mediated knockdown with the *NP2631*, *TH* and *pox9-1-6 GAL4* drivers, respectively. Fru^{C} expression in adjacent, GFP negative cells is unaffected. **C** Sine song production after Fru^{C} knockdown in dsx neurons (*dsx-GAL4*), P1 (*NP2631),* aSP4 *(TH-GAL4),* or vAB3 *(pox9-1-6-GAL4)*. n=57-60 flies per genotype. **D** IPI distribution of pulse song of the experimental flies represented in C. n=57-60 flies per genotype, 50-1500 IPIs per fly. C, D: Kruskal-Wallis non parametric ANOVA, followed by Dunn's multiple comparisons test, ***p<0.0001, *p<0.05. Box and whisker plots show 10, 25, 50, 75 and 90 percentiles. Yellow bars represent knockdown experiments, white bars controls.

	Pupa CNS	Adult CNS
**A**	235 ± 47 (15%)	354 ± 78 (22%)
**B**	159 ± 54 (10%)	175 ± 44 (11%)
**C**	49 ± 12 (3%)	94 ± 21 (6%)
**A, B**	124 ± 43 (8%)	76 ± 23 (5%)
**A, C**	44 ±13 (3%)	28 ± 12 (2%)
**B, C**	235 ± 53 (15%)	277 ± 17 (17%)
**A, B, C**	728 ± 76 (46%)	604 ± 69 (38%)
**total**	1573 ± 89 (n=5)	1607 ± 123 (n=5)

Table S1, related to Figure 1

Fru isoform expression in the pupa and adult

Number of cells expressing exclusively one, two or all three isoforms (mean ± standard deviation, n=5 animals) and percentages of the total Fru positive cells in the CNS of 2d old pupa and 8d old adults, respectively. Neurons in the mushroom body are not included.
